# Patient-Derived Xenografts and Organoids Recapitulate Castration-Resistant Prostate Cancer with Sustained Androgen Receptor Signaling

**DOI:** 10.3390/cells11223632

**Published:** 2022-11-16

**Authors:** Annelies Van Hemelryk, Ingrid Tomljanovic, Corrina M. A. de Ridder, Debra C. Stuurman, Wilma J. Teubel, Sigrun Erkens-Schulze, Esther I. Verhoef, Sebastiaan Remmers, Amrish J. Mahes, Geert J. L. H. van Leenders, Martin E. van Royen, Harmen J. G. van de Werken, Magda Grudniewska, Guido W. Jenster, Wytske M. van Weerden

**Affiliations:** 1 Department of Urology, Erasmus University Medical Center, Dr. Molewaterplein 40, 3015 GD Rotterdam, The Netherlands; 2 GenomeScan B.V., Plesmanlaan 1/D, 2333 BZ Leiden, The Netherlands; 3 Department of Pathology, Erasmus University Medical Center, Dr. Molewaterplein 40, 3015 GD Rotterdam, The Netherlands; 4 Cancer Computational Biology Center, Erasmus University Medical Center, Dr. Molewaterplein 40, 3015 GD Rotterdam, The Netherlands; 5 Department of Immunology, Erasmus University Medical Center, Dr. Molewaterplein 40, 3015 GD Rotterdam, The Netherlands

**Keywords:** castration-resistant prostate cancer, patient-derived xenografts, organoids, androgen receptor, drug testing

## Abstract

Castration-resistant prostate cancer (CRPC) remains an incurable and lethal malignancy. The development of new CRPC treatment strategies is strongly impeded by the scarcity of representative, scalable and transferable preclinical models of advanced, androgen receptor (AR)-driven CRPC. Here, we present contemporary patient-derived xenografts (PDXs) and matching PDX-derived organoids (PDXOs) from CRPC patients who had undergone multiple lines of treatment. These models were comprehensively profiled at the morphologic, genomic (*n* = 8) and transcriptomic levels (*n* = 81). All are high-grade adenocarcinomas that exhibit copy number alterations and transcriptomic features representative of CRPC patient cohorts. We identified losses of *PTEN* and *RB1*, *MYC* amplifications, as well as genomic alterations in *TP53* and in members of clinically actionable pathways such as AR, PI3K and DNA repair pathways. Importantly, the clinically observed continued reliance of CRPC tumors on AR signaling is preserved across the entire set of models, with *AR* amplification identified in four PDXs. We demonstrate that PDXs and PDXOs faithfully reflect donor tumors and mimic matching patient drug responses. In particular, our models predicted patient responses to subsequent treatments and captured sensitivities to previously received therapies. Collectively, these PDX-PDXO pairs constitute a reliable new resource for in-depth studies of treatment-induced, AR-driven resistance mechanisms. Moreover, PDXOs can be leveraged for large-scale tumor-specific drug response profiling critical for accelerating therapeutic advances in CRPC.

## 1. Introduction

Prostate cancer (PCa) growth and progression are driven by the androgen receptor (AR) signaling pathway. Consequently, systemic suppression of androgens through chemical or surgical castration forms the basis for treating advanced PCa. However, tumor response to castration is only temporary, and patients inevitably develop lethal castration-resistant prostate cancer (CRPC). The clinical management of CRPC is challenging and currently involves multiple lines of treatment, which leads to treatment-induced genomic alterations and selective survival of highly resistant subclones [1,2]. Importantly, even at this lethal stage, the vast majority of CRPC tumors remain reliant on a reactivated and often highly augmented AR signaling, with AR pathway aberrations detected in over 80% of CRPC patients [1,3,4]. Furthermore, a large proportion of these patients harbor additional, potentially targetable, alterations (such as those involving the DNA damage response or PI3K pathway) [3,4]. This highlights the heterogeneity, but also the therapeutic opportunities for patients with castration-resistant disease. Exploring these opportunities and improving current therapeutic strategies is crucial to maximize patient survival but requires comprehensive testing on robust and representative AR-positive (AR+) preclinical models.

Patient-derived xenografts (PDXs) display the closest resemblance to actual patient tumors. These in vivo models capture the pathologic, molecular and genetic features of the original human tumor, whose microenvironment is mimicked by the murine stroma and vasculature [5]. A substantial collection of PCa PDXs has been assembled over the last few decades [6,7,8,9,10]. However, only limited numbers of CRPC PDXs from patients receiving contemporary therapies or those subjected to multiple lines of treatment have been generated so far [10,11,12], hampering the development of innovative therapeutic approaches for this growing patient population. The evolving and heterogeneous clinical CRPC landscape thus necessitates a continuous expansion of the currently available PDX series.

Despite their utmost importance, PDXs are restricted to low-throughput assessments. They do, however, provide an indefinite source of tumor material to generate organoids [13,14]. These 3D in vitro models are supposed to maintain original tumor characteristics, architecture and heterogeneity [15], a prerequisite that has not been fulfilled by PCa cell lines. The use of CRPC PDX-derived organoids (PDXOs) allows for a drastic scale up of preclinical testing capacities and might substantially accelerate clinical translation.

Here, we introduce a new set of AR+ CRPC models obtained from heavily pretreated patients, including PDXs and matching PDXOs. All models demonstrated sustained AR signaling and preservation of key phenotypic, transcriptomic and genetic features of original tumors. Importantly, both PDXs and PDXOs faithfully reproduced matching patient drug responses to standard of care chemotherapeutics and showed utility in predicting the sensitivity to AR signaling inhibitors.

## 2. Materials and Methods

### 2.1. Patient Samples and PDX Development

Tumor samples were procured from PCa patients undergoing surgery or (metastatic) biopsy at any of the hospital partners within the Rotterdam region (Supplementary Table S1). Written informed consent was obtained before sampling. The Erasmus MC Medical Research Ethics Committee assessed this research as not being subject to the Dutch Medical Research Involving Human Subjects Act (WMO) and as such does not require approval from a local ethics committee (MEC-2013-240). Samples and clinical data were handled in accordance with the Code of Conduct for Responsible Use.

Patient samples were transferred on ice immediately after surgery and macrodissected upon arrival in the laboratory. Small tumor fragments (50 mm^3^) were implanted subcutaneously at both scapular regions of six- to eight-week-old NMRI nu/nu male mice (*n* = 2–4, Taconic, Ry, Denmark), under isoflurane anesthesia and analgesia with carprofen. Neighboring tissue was formalin fixed and paraffin embedded (FFPE), and snap frozen for −80 °C storage. Mice were monitored for at least six months and checked weekly for tumor growth. 

PDX tumors are maintained by serial mouse transplantations and typically transplanted into new recipients (*n* = 2–4) when tumors reach a volume of 1500 mm^3^. At each mouse-to-mouse transplantation, representative tumor fragments are FFPE and snap frozen for quality control. PDX authenticity was confirmed by short tandem repeat (STR) analysis (PowerPlex 16 System, Promega, Madison, WI, USA). The absence of Epstein-Barr virus-associated lymphomagenesis was confirmed by qPCR [16] in all PDXs. 

### 2.2. Generating and Culturing PDXOs

To initiate PCa PDXOs, we developed a standard operating protocol adapted from Drost et al. [17] and Beshiri et al. [13]. PDX tumors were removed when reaching a volume of 1000–1500 mm^3^; they were minced using scissors and next digested enzymatically for 1 h at 37 °C using collagenase A (2 mg/mL, Roche Diagnostics, Mannheim, Germany, cat. no. 11088793001) in adDMEM/F12^+++^ supplemented with 10 µM Y-27632 (Supplementary Table S2), with mixing every 15 min. We performed an additional digestion of 10 min in TrypLE Express (Thermo Fisher Scientific, Waltham, MA, USA, cat. no. 12605028) containing 10 µM Y-27632, with mixing every 5 min. Digested tissue was washed in adDMEM/F12^+++^ with Y-27632 and passed through a 100 µm cell strainer (Falcon, Corning, NY, USA, cat. no. 352360). Filtered single cells and small cell clumps were centrifuged and resuspended in ice-cold adDMEM/F12^+++^ complemented with a synthetic, thermo-sensitive, hydrogel (Noviogel-P5K, Noviocell, Sopachem, Ede, The Netherlands) at a medium-hydrogel ratio of 64.5: 35.5. The suspension was dispensed as 30 µL droplets in pre-warmed 24-well plates (Costar, Corning, cat. no. 3527) and overlaid with culture medium.

For all processed PDXs, three different organoid growth media were evaluated: prostate cancer organoid medium (PCOM) [17], adjusted prostate cancer organoid medium (APCOM) [18] and prostate growth medium (PGM) [19]. Different media compositions are listed in Supplementary Figure S1 and Supplementary Table S2. Culture medium was replaced every three to four days. We visually monitored organoid growth on a daily basis and passaged the organoids when sufficient density was reached. 

### 2.3. Formalin Fixation and Paraffin Embedding, Hematoxylin and Eosin Staining, Immunohistochemistry

Patient and PDX tumors were fixed with 4% paraformaldehyde at 4 °C for several days. Organoids were fixed within the intact hydrogel dome for two hours at 37 °C. Prior to paraffin embedding, the entire dome was embedded in 4% agarose in PBS. FFPE samples were sliced into 4 µm-thick sections. Histological tumor architecture was examined by hematoxylin and eosin staining and assessed by an experienced genitourinary pathologist. Immunohistochemistry was performed according to standard methods. In short, endogenous peroxidase activity was blocked with 0.3% hydrogen peroxide in PBS, followed by heat-induced antigen retrieval and blocking of nonspecific binding with 5% bovine serum albumin. Antibody details, including dilutions for tissues and organoids, are provided in Supplementary Table S3. For negative controls, primary antibodies were omitted. Expression was visualized with hydrogen peroxide/DAB (REAL EnVision Detection System, Dako, Santa Clara, CA, USA, cat. no. K5007) and counterstaining was performed with hematoxylin. Images were acquired with a Nikon Eclipse TS2 microscope equipped with 4×, 10× CFI Achro brightfield objectives and a 20× Fluor ELWD objective, a DS-Fi3 camera and NIS-Elements imaging software (Minato, Tokyo, Japan).

### 2.4. DNA and RNA Isolation

Snap frozen patient and PDX tumor tissues were homogenized using an Ultra-Turrax T25 (Janke & Kunkel, Staufen, Germany), after which total DNA and RNA were isolated with the AllPrep DNA/RNA Mini Kit (Qiagen, Germantown, MD, USA, cat. no. 80284) according to the manufacturer’s protocol. Early generation PDXOs were retrieved from the hydrogel by carefully dissolving the Noviogel dome with ice-cold PBS. Organoid RNA isolation was performed as described previously [18] using the AllPrep DNA/RNA Micro Kit (Qiagen, cat. no. 80284), according to the manufacturer’s protocol. 

An overview of DNA and RNA datasets generated and used in this study is provided in Supplementary Table S4.

### 2.5. DNA Genotyping and Copy Number Analysis

DNA genotyping was performed for PDX tumors, using the Infinium Global Screening Array 24v3 (Illumina, Hayward, CA, USA). B-allele frequency and log R ratio values were extracted using Illumina GenomeStudio 2.0 software and parsed into R for further analyses. Allele-specific copy number and tumor ploidy were estimated using ASCAT v2.5.2 [20] for all PDXs except PC2412, where the algorithm failed due to sample complexity. Copy number calling was based on ASCAT output transformed as the log_2_ (total copy number/tumor ploidy). All homozygous deleted segments were assigned a log_2_ value of −3. For selected genomic regions (22.2–101.08 Mb), the mean log_2_ value of segments overlapping the region was used for calling gains and losses. For individual genes, the segment with the lowest copy number was taken as representative of the gene in case of overlap with multiple segments. Amplified segments were identified as having log_2_ values > 0.1, while losses were defined by a threshold of <−0.1 [21]. Copy number similarity scores between samples were calculated based on all segments using the calculateOverlapMetric function and the Sorensen method in *CNVMetrics* v1.0.0 [22].

### 2.6. RNA Sequencing and Data Processing

RNA libraries were constructed using the NEBNext Ultra II Directional RNA Library Prep Kit for Illumina (New England BioLabs, Ipswich, MA, USA, cat. no. E7760S/L) according to the manufacturer’s instructions. In brief, mRNA was isolated using oligo-dT magnetic beads, followed by RNA fragmentation, cDNA synthesis, adapter ligation and PCR amplification of the cDNA library. RNA sequencing was performed to obtain 20 million reads per sample using the Illumina NovaSeq 6000, yielding 150 bp paired-end reads. Raw FASTQ files underwent the following steps: adapter trimming using ‘Trimmomatic’ v0.30; alignment to GRCh37.75 human reference (and GRCm38.p4 mouse reference for PDXs and PDXOs) with TopHat v2.0.14; human/mouse reads disambiguation using Disambiguate 1.0 [23]; gene level raw counts calculation using ‘HTSeq’ version 0.9.1 and GRCh37.75 (and GRCm38.p4 for xenografts) annotation files (.gtf) for human and mouse reads, respectively. 

### 2.7. Pathway Analyses

Pathway-level consistency was assessed by rank-based single-sample gene set scoring performed with the *singscore* package v1.13.1 on the cancer hallmarks (H) subset of the Molecular Signatures Database (MSigDB) v7.4 [24,25]. To identify cancer hallmarks differing significantly between hormone sensitive and castration-resistant PDXs, gene set enrichment analysis (GSEA) [25] was performed using the *clusterProfiler* [26] package v3.18.1 and the Benjamini-Hochberg correction with a significance threshold of 0.05. Gene set variation analysis (GSVA) was applied to vst counts of the cancer hallmarks gene sets and the TMPRSS2:ERG signature gene set retrieved from MSigDB v7.4 using the *GSVA* package [27] v1.38.2. Pathway activity *t*-distributed stochastic neighbor embedding (*t*-SNE) analysis was performed on a matrix of GSVA scores of the cancer hallmark gene sets using the *Rtsne* package v0.15 [28].

### 2.8. AR Activity and Neuroendocrine Signature Scoring 

AR activity scores were estimated using a reference gene expression vector for a previously described 30-gene signature [29]. The reference was constructed using mean mRNA transcript abundances from a set of 60 metastatic CRPC adenocarcinomas with an active AR signaling [30]. The score was then quantified as the Pearson correlation coefficient between the reference vector and the expression vector for each individual sample. All expression units were in log_2_(FPKM + 1). The neuroendocrine (NE) signature score was calculated analogously based on a set of 70 genes reported by Beltran et al. [31]. Samples with a score of ≤0.25 were defined as having low AR signaling, while a NE signature score ≤0.4 was used to distinguish adenocarcinomas from NE PCa tumors [31]. 

### 2.9. AR Splice Variant Identification

Quantitative RT-PCR was performed as described previously [32] to assess expression levels of full-length *AR* and *AR* variants (*AR-V1*, *V3*, *V7*, *V9*, *V12*). Primers used are listed in Table S5. Expression was normalized to *GAPDH* using the 2^−ΔCt^ method. For each AR-V assessed by RT-PCR, the percentage spliced in (PSI) was also quantified from the RNA sequencing data using a custom R script.

### 2.10. Quantification of Prostate-Specific Antigen (PSA) in Organoid Medium

PSA concentration in the organoid medium was quantified with a human PSA enzyme-linked immunosorbent assay (ELISA) kit (Abnova, Taipei City, Taiwan, cat. no. KA0208) according to the manufacturer’s protocol. Absorbance was measured at 450 nm using a microtiter plate reader (Bio-rad 550, Hercules, CA, USA) and PSA concentration (ng/mL) was calculated from a linear standard curve of supplied reference standards.

### 2.11. Assessment of Murine Contamination in PDXO Cultures

Organoids were cultured in black 96-well CellCarrier Ultra plates suitable for high content imaging (Perkin Elmer, Hamburg, Germany, cat. no. 6055302) and incubated with Hoechst 33342 (2 µg/mL, Invitrogen, Thermo Fisher Scientific, cat. no. H3570) for 3 h prior to imaging with the Opera Phenix High Content Screening System (Perkin Elmer) equipped with a 40× water immersion objective and a 16-bit sCMOS 4 Megapixel camera. Hoechst was excited with a 405 nm solid-state laser line and detected at 435–480 nm wavelength range. Organoids were imaged in the hydrogel dome, using 100 plane Z-stacks with a step size of 2 µm between planes. Nuclei in organoids of at least 50 µm in diameter were manually assessed for the presence of chromocenters, that are visible as high-intensity nuclear foci.

### 2.12. Animal Experiments

Animal housing, animal care and all animal experiments were performed in accordance with the Dutch Experiments on Animal Act and approved by the Animal Tests Committee under license number AVD101002017867. Sample size was determined by power analysis based on preliminary data.

For in vivo assessment of androgen response, 18 six-week-old NMRI nu/nu intact male mice per PDX were subcutaneously implanted with 50 mm^3^ tumor fragments. Tumors were measured twice a week using calipers and tumor volume was calculated with the equation (π/6)*(tumor length × tumor width)^3/2^. When tumors reached a volume of ~300 mm^3^, mice were allocated to two groups to undergo either surgical castration (bilateral orchiectomy) or sham castration under ketamine/medetomidine anesthesia (75 mg/kg and 1 mg/kg) and analgesia with carprofen (5 mg/kg). Castrated mice were again split into two groups at 30 days after castration, to receive either a silastic implant with 40 mg testosterone (AppliChem, Darmstadt, Germany, cat. no. A0671) or an empty (placebo) implant. Tumor volume was followed for a maximum of 60 days after castration or until tumors exceeded a volume of 1500 mm^3^.

To assess in vivo taxane sensitivity, 24 six-week-old NMRI nu/nu intact male mice were transplanted per PDX. When tumor volumes reached ~300 mm^3^, animals were divided into three groups and administered a single intravenous injection of either docetaxel (33 mg/kg; Sanofi, Paris, France), cabazitaxel (33 mg/kg; Sanofi) or sodium chloride (placebo). Tumor volumes were measured twice a week and animals were euthanized when volumes exceeded 1500 mm^3^ or when a maximum follow-up of 90 days after taxane administration was reached. 

### 2.13. Organoid Drug Assays

PDX tumors were processed to initiate PDXO cultures, as described above. Organoids were allowed to preassemble in 24-well plates and harvested after 7 to 14 days by dissolving the Noviogel dome with ice-cold medium. Organoids were filtered using a 100 µm cell strainer and plated at a density of 5000–10,000 organoids per 8 µL Noviogel domes in white clear bottom 96-well plates (Costar, Corning, cat. no. 3610). After four days in APCOM, the medium was removed and replaced by APCOM containing the appropriate drug concentration. Organoids were incubated with a dose range of docetaxel, cabazitaxel (Sanofi) or enzalutamide (Axon Medchem, Groningen, the Netherlands, cat. no. 1613) for 10 days. Each treatment plate contained positive controls (1 µM staurosporine; Selleckchem, Houston, TX, USA, cat. no. S1421) and negative controls (solvents). On the day of drug administration (day 0) and after 10 days incubation, organoid viability was measured with CellTiter-Glo 3D (Promega, cat. no. G9681). An increase in organoid viability of the untreated controls of at least 1.5 between day 0 and day 10 was considered as sufficient growth and was the main inclusion criterion for any further analyses. Additionally, the following quality control metrics were verified for each dose-response plate: coefficient of variation (CV) < 0.22, Z-factor > 0.4 and, if applicable, DMSO-effect 0.8–1.2 [33]. Quality criteria were met for all included experiments, except for CV and Z-factor criteria in PC2412 PDXOs due to the outgrowth of large organoids.

### 2.14. Statistical Analysis

R software v4.1.0 [34] and GraphPad Prism 9 (GraphPad, San Diego, CA, USA) were used for statistical analyses. 

The baseline RNA sequencing dataset (Supplementary Table S4) was inspected with unsupervised hierarchical clustering of sample-to-sample Euclidean distances calculated using vst counts of all features, based on which four samples with transcriptomic profiles of low similarity to the rest of the dataset were excluded from downstream analyses (Supplementary Figure S2A). Of these, the PC2400 patient sample was specifically shown to differ from other patient samples based on expression of the *MSMB* gene using principal component analysis on 1000 most variable features (Supplementary Figure S2B), indicating that this sample consisted of benign prostate tissue [35]. After exclusion, the RNA sequencing dataset consisted of four patient samples, 34 PDX samples and 39 PDXO samples. 

For AR score association testing, the Kruskal-Wallis test was performed in R. Average survival benefit of docetaxel or cabazitaxel compared to placebo was analyzed using the restricted mean survival time [36]. The level of statistical significance was set at 0.05 and a Bonferroni correction was applied for multiple testing, unless otherwise specified. Dose-response curves were generated using the nonlinear regression curve fit method (4 parameters) in GraphPad Prism. Data are plotted as a mean with error bars of SD or SEM, as specified in the figure legends. For in vivo and in vitro experiments, the number of technical and biological replicates is indicated in the corresponding figure legends.

### 2.15. Data Visualization 

R software v4.1.0 [34] and GraphPad Prism 9 (GraphPad) were used for data visualization. Schematic figures were created with BioRender.com. Co-clustering with the patient cohort from Labrecque et al. [30] was visualized with the *pheatmap* package v1.0.12 [37] through hierarchical clustering of sample-to-sample Euclidean distances using 21,685 features, after batch effect correction for cohort of origin and sample type with the ComBat function in the *sva* package v3.38.0 [38]. Gene expression heatmaps were constructed using vst counts and the *pheatmap* package v1.0.12 [37]. *t*-SNE was used for unsupervised dimensionality reduction to visualize underlying clusters in the dataset and inspect model consistency. The algorithm was applied to vst counts of 2500 most variable genes with the *Rtsne* package v0.15 [28]. PCA plots were visualized using the *PCAtools* package v2.2.0 [39]. Array data tracks were visualized using the *karyoploteR* v1.16.0 [40] and *CopyNumberPlots* v1.6.0 [41] packages.

## 3. Results

### 3.1. PCa PDXs Resemble Phenotypic and Genotypic Characteristics of Patient Tumors

**Figure 1:**
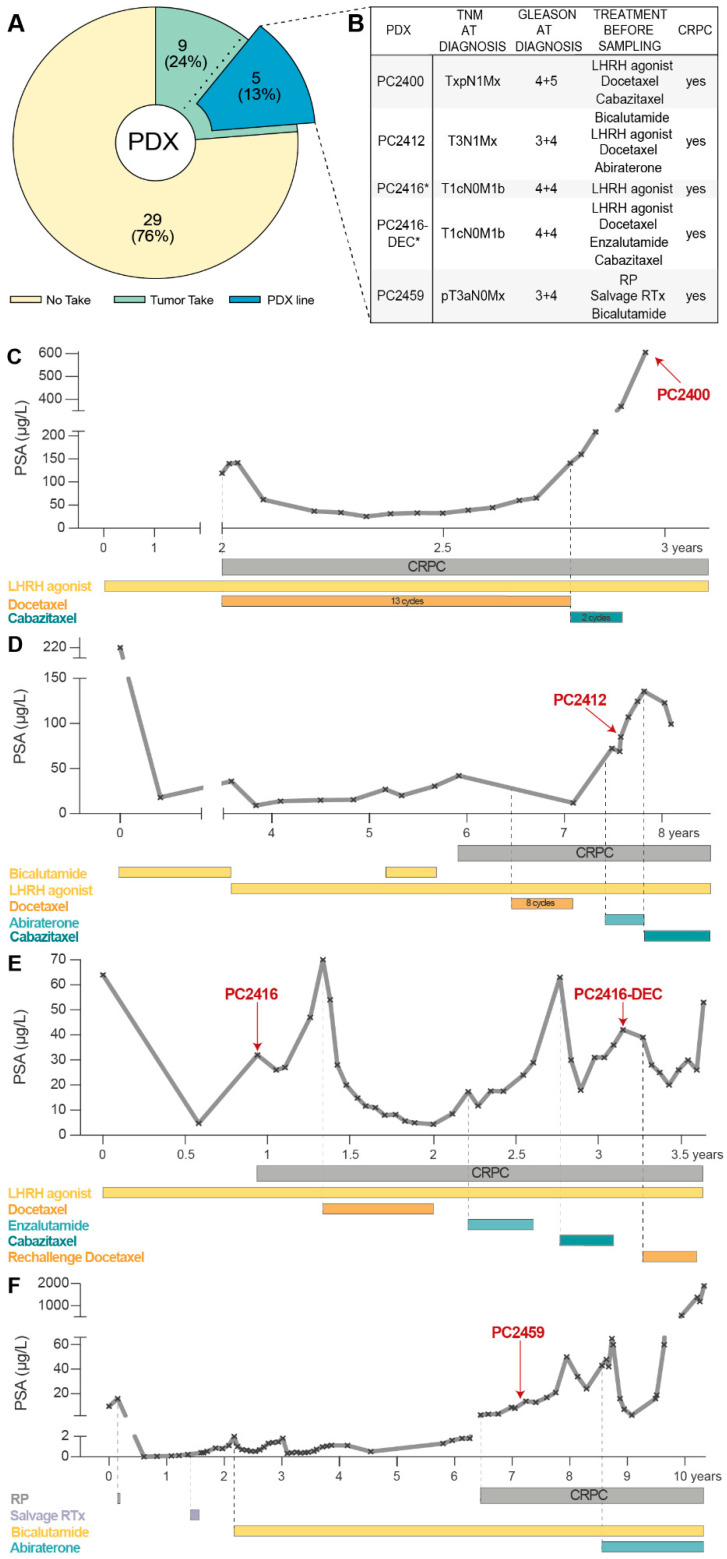
Development of CRPC PDXs. (**A**) Pie chart of PCa samples subcutaneously implanted in intact male nude mice to generate PDXs. No take (yellow) indicates absence of tumor outgrowth during a monitoring period of six months. Tumors take (green) represents successful engraftment followed by one to two mouse passages. PDX line (blue) indicates established PDXs through continuous mouse-to-mouse propagation. (**B**) Clinical and pathological patient characteristics for established PDXs. Treatment history prior to sampling is provided, including local treatments (radical prostatectomy (RP) and radiation therapy (RTx)), and systemic treatments (luteinizing hormone-releasing hormone (LHRH) agonists, chemotherapy, and AR signaling inhibitors). * Indicates identical patient origin. (**C**–**F**) Treatment timelines of the patients that gave rise to PC2400 (**C**), PC2412 (**D**), PC2416 and PC2416-DEC (**E**), and PC2459 (**F**) PDXs, depicted as PSA response from time of diagnosis (day 0) to death. Time point of tissue sampling for PDX engraftment is indicated by a red arrow.

Between April 2012 and December 2018, 38 PCa samples from 37 patients were implanted subcutaneously into intact male nude mice. The vast majority of samples (82%, Supplementary Table S1) originated from advanced PCa patients undergoing a transurethral resection of the prostate (TURP). Nine samples (eight TURP specimens and one metastatic lymph node sample) showed evidence of successful tumor engraftment and could be transplanted into other recipient male mice; five of these (all TURP samples) resulted in long-term PCa PDX lines that are being serially propagated from mouse to mouse (Figure 1A). Complementing our existing PDX panel [6,7,42], these five new PCa PDXs (PC2400, PC2412, PC2416, PC2416-DEC and PC2459) span diverse patient profiles of castration-resistant disease. Clinical and pathological patient characteristics are listed in Figure 1B, patient histories are depicted in Figure 1C–F. Notably, PDXs PC2416 and PC2416-DEC were derived from the same patient at different disease stages, i.e., PC2416 at time of biochemical recurrence under androgen deprivation therapy (ADT) and PC2416-DEC after subsequent systemic treatments with docetaxel (D), enzalutamide (E) and cabazitaxel (C), respectively (Figure 1E). 

**Figure 2:**
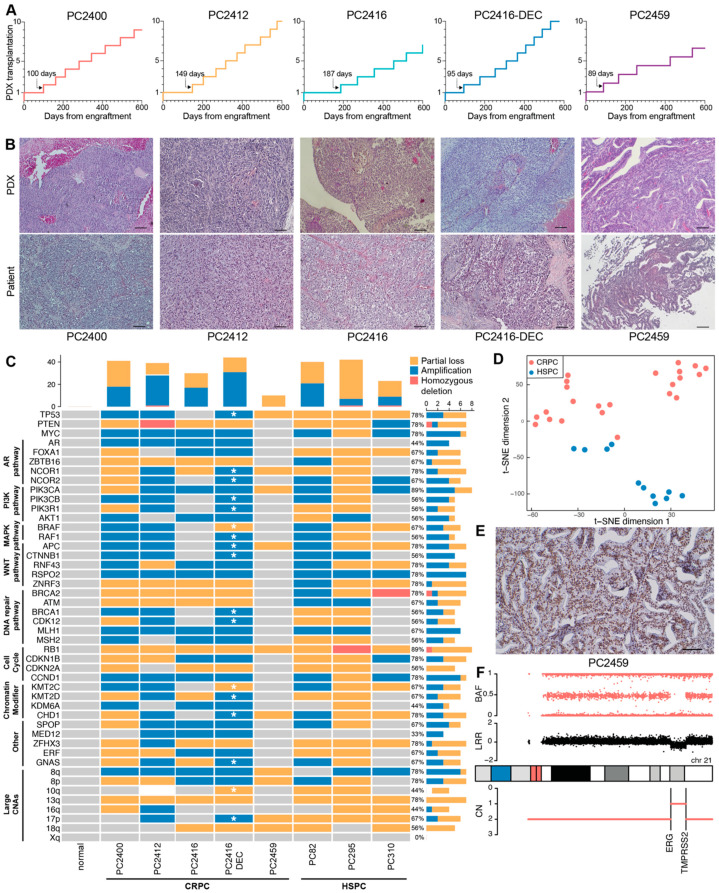
Characteristics of CRPC xenografts. (**A**) Growth trajectories of PDXs in the first 600 days after engraftment. Each step marks a mouse-to-mouse transplantation. Time from tumor engraftment to first mouse-to-mouse transplantation is indicated by an arrow. (**B**) Histological architecture of PDXs (top panel) and original patient tumors (bottom panel), examined by hematoxylin and eosin staining. Scale bars equal 100 µm. (**C**) CNA profiles of CRPC PDXs (PC2400, PC2412, PC2416, PC2416-DEC and PC2459), HSPC PDXs (PC82, PC295 and PC310) and a normal prostate tissue control from an unmatched source, across a subset of genes and genomic regions recurrently altered in CRPC. Grouping per biological pathway. Amplifications (blue), homozygous deletions (red), partial losses (yellow) and unaltered regions (grey) are indicated based on ASCAT analysis of SNP array data (*n* = 1 for each PDX). ASCAT analysis failed for PC2412 PDX due to sample complexity. All PC2412 CNA calls were determined manually except for the region 10q with complex rearrangements (white). CNA frequency bar plots are provided for each PDX (top panel) and each genomic region (right panel). Additional alterations present in PC2416-DEC PDX, as compared to PC2416 PDX are marked with a white asterisk. (**D**) *t*-SNE plot of pathway activity in CRPC PDXs (PC2400 (*n* = 5), PC2412 (*n* = 5), PC2416 (*n* = 5), PC2416-DEC (*n* = 5) and PC2459 (*n* = 3); red) and HSPC PDXs (PC82 (*n* = 2), PC295 (*n* = 2), PC310 (*n* = 7); blue), based on GSVA scores for 50 MSigDB cancer hallmark gene sets, using RNA sequencing data. (**E**) Nuclear ERG staining in PC2459 PDX. Scale bar indicates 100 µm. (**F**) Copy number profile of chromosome 21 in PC2459 PDX, displaying the *ERG* and *TMPRSS2* genes on 21q22.2-3. BAF: B allele frequency; LRR: log R ratio; CN: total copy number.

Time from tumor implantation to first mouse-to-mouse transplantation varied between three to six months (median: 100, IQR: 76 days; Figure 2A). Initial PDX generations (≤10 mouse passages) typically present slower and inconstant growth rates (Supplementary Figure S3A,B). Beyond mouse passage 10, growth properties become more consistent, with mean time between passages ranging from 36 to 46 days and current doubling times ranging from 12 to 25 days. 

Even after these long-term serial passages, PDXs retained the morphological growth patterns of corresponding patient tumors at the time of tissue sampling (Figure 2B). All five pairs of matched PDX-patient tumors were classified as high-grade adenocarcinomas: four of them lack or sparsely exhibit glandular differentiation, whereas PC2459 PDX and matching patient tumor display a cribriform growth pattern. Each PDX has its own unique short tandem repeat (STR) profile that matches the original patient tissue (92.6–100% match), with PDXs PC2416 and PC2416-DEC exhibiting identical profiles as they originated from the same patient (Supplementary Table S6). Importantly, STR profiles were conserved between early and late PDX generations (95–100% match). 

We next profiled the new CRPC PDXs against a comprehensive set of copy number altered genes and genomic regions identified in large CRPC patient cohorts [3,4] and compared these CRPC PDXs with three treatment-naïve, hormone sensitive PCa (HSPC) PDXs from our existing PDX panel. These HSPC PDXs were derived from rather aggressive primary tumors (PC82 [42] and PC310 [6]) and from a metastatic lymph node lesion (PC295 [6]) and, as such, are expected to carry a high burden of copy number alterations (CNAs) [43,44]. Across all eight PDXs, we confirmed the presence of common PCa-associated genomic alterations [3,4,43], including losses and gains of *TP53*, *PTEN*, *RB1* and *MYC* as well as large chromosomal aberrations on 8p, 8q, 10q, 13q, 16q, 17p and 18q (Figure 2C). Total copy number profiles of sequential PDXs PC2416 and PC2416-DEC were highly similar, sharing the majority of altered regions across the entire karyotype (similarity score 0.63). Yet, PDX PC2416-DEC harbored a number of additional CNAs annotated by an asterisk in Figure 2C.

Notably, *RB1* loss was present in all PDXs, with a homozygous deletion in PC295. *PTEN* copy number was altered in all PDXs with the exception of PC2459, while a homozygous *PTEN* deletion was identified in PC2412. Furthermore, we confirmed the presence of a previously identified homozygous *BRCA2* deletion in PC310 [45]. *AR* amplification, an event occurring in a large portion of CRPC tumors but rarely in treatment-naïve PCa [3,4,43], was identified in four out of five CRPC PDXs and was absent in all three HSPC PDXs. 

Additionally, our PDX cohort harbors a range of genomic alterations in members of clinically actionable pathways previously reported to be affected in PCa [3,43], including the AR, PI3K, WNT, MAPK/ERK, DNA repair pathways and the cell cycle, which opens opportunities for preclinical PCa drug testing. Identification of these driver genomic alterations in our HSPC models (PC82, PC295 and PC310) confirms their origin from more aggressive primary tumors. However, CRPC and HSPC PDXs clearly form distinct groups, visible at the transcriptomic level (Figure 2D), where they exhibit a significantly different enrichment of 14 cancer hallmark gene sets, such as epithelial mesenchymal transition (EMT) and hypoxia (Supplementary Figure S3C).

Within our CRPC PDX cohort, only PC2459 PDX revealed nuclear ERG expression upon immunohistochemical analysis, indicative of the *TMPRSS2-ERG* gene fusion being present solely in this PDX (Figure 2E and Supplementary Figure S3D). Inspection of SNP array data indeed confirmed a decrease in total signal intensity (log R ratio; LRR) between the *ERG* and *TMPRSS2* genes on 21q22.2-3 (Figure 2F), pointing towards the existence of the *TMPRSS2-ERG* fusion gene. While no *TMPRSS2-ERG* gene fusion was detected in the other CRPC PDXs, we confirmed the presence of complex rearrangements at this locus for HSPC PDXs PC82, PC295 and PC310, as described previously [46] (Supplementary Figure S3E). For all PDXs, the *TMPRSS2-ERG* gene fusion status was further corroborated at the transcriptomic level, showing high *ERG* expression and positive enrichment of the *TMPRSS2-ERG* fusion gene signature only in affected PDXs (Supplementary Figure S3F). Altogether, our PCa PDX panel comprises diverse phenotypic and genotypic profiles representative of actual patient tumors.

### 3.2. In Vitro PDXOs as Substitute Preclinical Models

**Figure 3:**
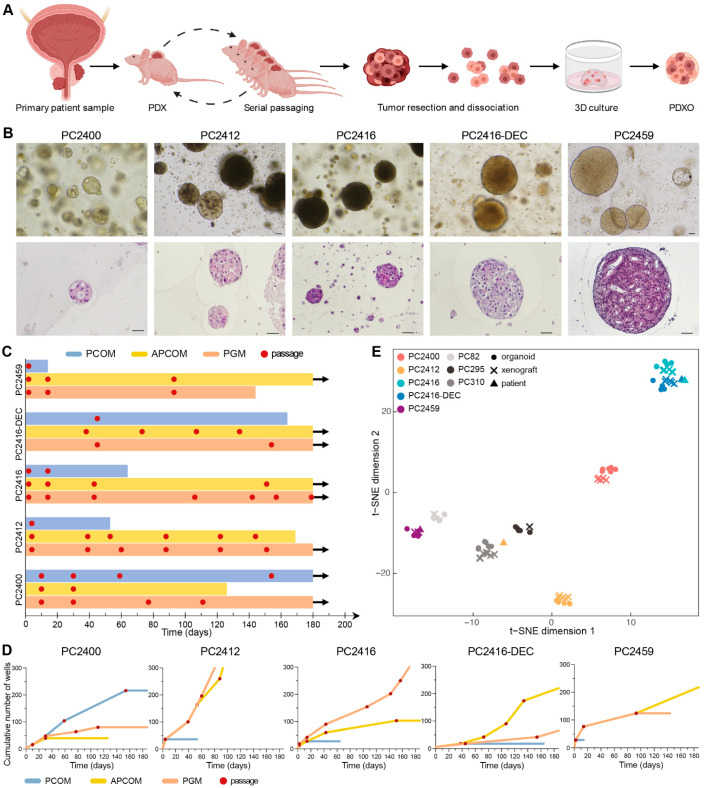
Establishment of PDXOs that reflect phenotypic and transcriptomic profiles of matching PDX and patient tumors. (**A**) Schematic workflow for creating PCa PDXs and PDXOs. Figure adapted from Van Hemelryk et al. [47]. (**B**) Top panel: Representative bright field images of PDXOs cultured in their optimal growth medium. Bottom panel: histological morphology of equivalent PDXOs, assessed by hematoxylin and eosin staining. Scale bars equal 50 µm. (**C**) Swimmer’s plot visualizing the number of days PDXOs could be viably maintained in each of the three distinct organoid culture media: PCOM (blue), APCOM (yellow) and PGM (orange). Organoid passages are marked with a red dot. Arrows indicate vital organoid culture beyond 180 days of in vitro propagation. (**D**) PDXO growth curves during long-term culturing, plotted as the cumulative number of wells over time and visualized up until six months after culture initiation. Each red dot represents a passage. (**E**) *t*-SNE plot based on gene expression profiles of original patient samples (*n* = 4), derived PDXs (*n* = 34) and PDXOs (*n* = 39), as quantified by RNA sequencing.

To generate PDXOs, we processed all five new PDXs along with seven previously established PDXs [6,7] (Figure 3A). Each PDX gave rise to PDXOs and allowed for early organoid passaging (Figure 3B and Supplementary Figure S4A). We systematically tested three different organoid growth media to initiate, expand and maintain PDXOs: prostate cancer organoid medium (PCOM) [17], adjusted prostate cancer organoid medium (APCOM) [18] and prostate growth medium (PGM) [19] (Supplementary Figure S1). Each PDX had its optimal medium, with PCOM being least successful overall (Figure 3C,D and Supplementary Figure S4B,C). We particularly focused on the new CRPC PDXs for continued organoid culturing; although PDXOs were viable for over six months (Figure 3C), the highest proliferation rates were observed in the first months after plating (Figure 3D and Supplementary Figure S4B). 

Early generation PDXOs were extensively assessed for their reliance on key phenotypic and molecular features of the original tumors. PDXOs originating from xenografts without glandular differentiation formed dense solid structures in vitro (Figure 3B), while PDXOs from PC2400 PDX assembled as hollow configurations with a subtle luminal differentiation similar to the respective patient and PDX tumor (Figure 2B and Figure 3B). Likewise, cribriform features present within PC2459 patient and PDX tumors were maintained in the corresponding PDXOs. Concordance of transcriptomic profiles between patients, PDXs and PDXOs further confirmed model consistency as models invariably clustered according to patient-of-origin (Figure 3E). Importantly, pathway activity also proved to be consistent between PDXs and their matching PDXOs (Supplementary Figure S3C). Overall, representative PDXOs can be established at high success rates once optimal culture conditions are defined per PDX.

### 3.3. AR Signaling Status of CRPC PDXs and PDXOs

**Figure 4:**
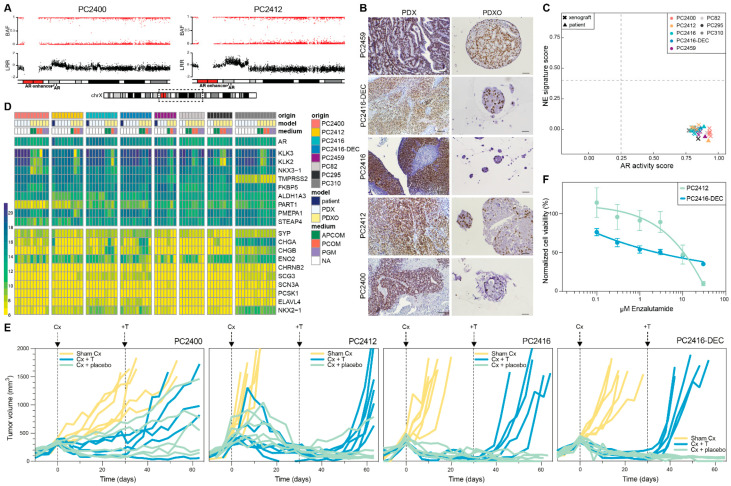
AR signaling status of CRPC PDXs and PDXOs. (**A**) B-allele frequency (BAF) and log R ratio (LRR) for the region of interest on chromosome Xq containing the *AR* gene and upstream *AR* enhancer, in PDXs PC2400 and PC2412. Dashed box on the chromosome X ideogram indicates the genomic region displayed above. (**B**) Nuclear AR staining in CRPC PDXs (left panel, scale bars 100 µm) and matching PDXOs (right panel, scale bars 50 µm). (**C**) Landscape plot of AR activity and NE signature scores in PDX (*n* = 34) and patient (*n* = 4) tumors. Thresholds separating samples with low versus high scores are marked with dashed lines. (**D**) Gene expression heatmap of a curated set of AR and NE marker genes shown across the entire dataset comprising original patient tumors (blue, *n* = 4), PDXs (white, *n* = 34) and PDXOs (yellow, *n* = 39) with different culture medium conditions (green: APCOM; red: PCOM; purple: PGM; white: not applicable (NA)). Expression levels are visualized as vst counts, based on RNA sequencing data. (**E**) Tumor growth responses after surgical castration (Cx) and testosterone supplementation (+T) in CRPC PDXs. When tumors passed a volume of 300 mm^3^ (day 0), mice underwent either Cx (blue and green; PC2400 *n* = 10, PC2412 *n* = 12, PC2416 *n* = 11, PC2416-DEC *n* = 10) or sham Cx (yellow; *n* = 6 for each PDX). Thirty days after Cx, mice were implanted with a testosterone pellet (blue; PC2400 *n* = 5, PC2412 *n* = 6, PC2416 *n* = 5, PC2416-DEC *n* = 5) or placebo pellet (green; PC2400 *n* = 5, PC2412 *n* = 6, PC2416 *n* = 6, PC2416-DEC *n* = 5). (**F**) Dose-response curves of PC2412 and PC2416-DEC PDXOs exposed to enzalutamide treatment. Dots mark mean and error bars SEM of three and four individual experiments (six technical replicates per condition) for PC2412 and PC2416-DEC PDXOs, respectively. Cell viability was normalized to vehicle (DMSO) controls.

We revealed several AR pathway gene members to be altered in copy number within our CRPC models, including *AR* itself, with *AR* amplification observed in four out of five CRPC PDXs (Figure 2C). Amplification of the *AR* gene is highly prevalent in CRPC patients, and often coincides with an amplification of the upstream located *AR* enhancer [4,48]. We indeed noted the amplified region to encompass not only the *AR* gene body, but also the *AR* enhancer in PDXs PC2400 and PC2412 (Figure 4A and Supplementary Figure S5A). Similar to previous observations [49], we noted elevated *AR* mRNA expression in several CRPC PDXs (Supplementary Figure S5B,C). Moreover, we identified co-expression of multiple AR variants in both HSPC and CRPC PDXs (Supplementary Figure S5D,E), in concordance with recent studies on PCa patient tumors [50,51].

Immunohistochemical analyses revealed nuclear AR and cytoplasmic PSA expression in PDXs and PDXOs (Figure 4B and Supplementary Figure S5F). In addition, elevated PSA levels were detected in the PDXO culture medium (containing 0.1 nM of R1881), even after long-term serial organoid passaging (Supplementary Figure S5G). Within one generation, PDXOs manifested rising medium PSA levels over time, reflecting an increase in the number of organoid cells (Supplementary Figure S5H). 

As these results indicate a functional AR signaling pathway both in vivo and in vitro, we further interrogated AR pathway activity based on a gene expression signature defining the pathway [29]. The presence of neuroendocrine (NE) transcriptional features was investigated alongside. All CRPC patient-PDX pairs presented an active AR signaling and were classified as non-NE (Figure 4C,D). Furthermore, their transcriptomic profiles showed the highest degree of similarity with AR+ adenocarcinomas when compared to an external cohort of CRPC patient tumors (*n* = 98; Supplementary Figure S6A). Notably, a group of PDXOs manifested lower AR scores (between 0.25 and 0.6; Figure S6B). This decreased AR activity was correlated with medium type, with the lowest scores observed for PCOM and highest for PGM (Supplementary Figure S6C,D). 

Surprisingly, in organoids derived from PC2416 PDX, AR staining was weak to absent (Figure 4B), and PSA secretion was low (Supplementary Figure S5G), despite its *AR* amplification and high *AR* mRNA expression (Supplementary Figure S5A–C)**.** For that reason, we verified the human origin of all long-term organoid lines and found PC2416 PDXO cultures to be grossly overgrown by murine organoids (Supplementary Figure S6E,F). This phenomenon recurred at all attempts to cultivate PDXOs from this specific PDX, independent of medium type. Strikingly, PGM appeared to support murine contamination also in long-term cultures of other PDXs, however without murine overgrowth (Supplementary Figure S6G).

To assess the growth dependence of our CRPC PDXs on the AR pathway, we evaluated the effect of surgical castration of the male host mice and subsequent testosterone supplementation. CRPC PDXs are sustained in intact male mice, in which circulating testosterone levels are assumed to mimic the low androgen levels present in CRPC patients under ADT. Castration of mice, however, causes testosterone levels to drop to abiraterone-treated human equivalents [52]. In three out of four CRPC PDXs, castration induced tumors to regress, followed by tumor relapse only after reintroducing testosterone (Figure 4E). PC2400-bearing mice demonstrated a mixed response, suggestive of a heterogeneous tumor cell population in this PDX. Collectively, these findings confirm sustained AR pathway activity in CRPC tumors, and they also imply a potential sensitivity to AR signaling inhibitors, such as abiraterone or enzalutamide.

We aimed to evaluate the sensitivity to enzalutamide using PDXOs from an enzalutamide-naïve (PC2412) and enzalutamide-pretreated patient (PC2416-DEC). Enzalutamide sensitivity was indeed corroborated in PC2412 PDXOs; while PC2416-DEC PDXOs demonstrated only a partial response (Figure 4F), in concordance with the actual patient response to enzalutamide (Figure 1E). High overexpression of *AR* and the presence of *AR* variants in PC2416-DEC PDX (Supplementary Figure S5B–E) might be underlying the observed enzalutamide resistance, as reported by others [11,48,49]. Overall, we demonstrate that our CRPC models remain largely dependent on AR signaling and carry important aberrations in the AR pathway, similar to the vast majority of CRPC patients.

### 3.4. PDXs and PDXOs Capture Patient Taxane Responses

**Figure 5:**
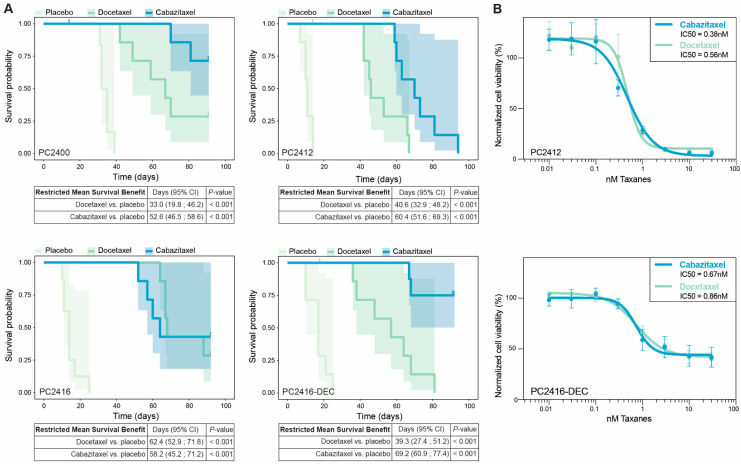
Responses of PDXs and PDXOs to taxane treatment. (**A**) Kaplan Meier plots depicting disease-specific survival of the tumor-bearing mice treated with placebo, docetaxel or cabazitaxel for PDXs PC2400 (placebo *n* = 6, docetaxel *n* = 7, cabazitaxel *n* = 8), PC2412 (placebo *n* = 7, docetaxel *n* = 8, cabazitaxel *n* = 7), PC2416 (placebo *n* = 8, docetaxel *n* = 7, cabazitaxel *n* = 7), and PC2416-DEC (placebo *n* = 7, docetaxel *n* = 8, cabazitaxel *n* = 8). Disease-specific survival was determined from the start of treatment (day 0) until tumor volumes exceeded 1500 mm^3^. Censored mice did not develop tumors of >1500 mm^3^ during a maximum follow-up of 90 days or died from other causes. Tables display restricted mean survival benefit of docetaxel/cabazitaxel over placebo. (**B**) Dose-response curves of PC2412 PDXOs (upper panel) and PC2416-DEC PDXOs (lower panel) exposed to cabazitaxel (blue) or docetaxel (green). Dots indicate mean and error bars SEM of three and four individual experiments, respectively (six technical replicates per condition). Cell viability was normalized to vehicle (ethanol) controls. Half-maximal inhibitory concentration (IC50) was calculated by sigmoidal-curve fitting.

After tissue sampling for PDX development, patients PC2412 and PC2416 underwent taxane-based chemotherapy (Figure 1D,E). This allowed us to assess the capacity of PDXs to retrospectively predict patient responses to subsequent treatments. Our consecutive PDX pair (PC2416 and PC2416-DEC) provided a unique resource for investigating this capacity, as the patient received docetaxel treatment after tissue sampling for PC2416 PDX and received a re-challenge with docetaxel after tissue sampling for PC2416-DEC PDX. At both disease stages, docetaxel therapy elicited a drop in PSA levels (Figure 1E). This repeated patient sensitivity to docetaxel was accurately reflected by the corresponding PDXs, as demonstrated by a significant survival benefit of docetaxel over placebo treatment in both (Figure 5A and Supplementary Figure S7). Interestingly, we noted a smaller survival benefit from docetaxel in PC2416-DEC-bearing mice compared to PC2416-bearing mice (39.3 days (95% CI = 27.4–51.2) versus 62.4 days (95% CI = 52.9–71.8)), mimicking the reduced patient response to the re-challenge with docetaxel (Figure 1E). Similarly, patient PC2412 underwent cabazitaxel treatment after tissue procurement (Figure 1D). Also in this case, the observed patient sensitivity to cabazitaxel was confirmed in its derived PDX, as shown by a survival benefit of 60.4 days (95% CI = 51.6–69.3) over placebo (Figure 5A and Supplementary Figure S7).

We also evaluated whether PDXs reflect patient sensitivities to previous treatments. Prior to tissue sampling, patient PC2412 received docetaxel therapy, leading to an evident PSA decline (Figure 1D). Only several months after premature treatment discontinuation, a relapse was observed. This indicates tumor sensitivity to the treatment, which was also observed when exposing PC2412-bearing mice to docetaxel (Figure 5A and Supplementary Figure S7). PC2400 patient, on the contrary, manifested PSA progression and an increase in metastatic burden during extensive docetaxel treatment (13th cycle) and subsequent cabazitaxel treatment (Figure 1C), indicating the occurrence of taxane resistance. Yet, we still observed response to taxanes in its derived PDX (Figure 5A and Supplementary Figure S7), that was generated posttreatment. These results suggest that tumor cells at the sampling site were still sensitive to taxanes at the time of disease progression. 

To assess whether PDXO drug responses mirror matching PDX and patient responses, we exposed PC2412 and PC2416-DEC PDXOs to relevant taxanes. For both docetaxel and cabazitaxel, we observed a concentration-dependent decrease in organoid viability (Figure 5B), proving organoid sensitivity to both chemotherapeutic drugs. We, however, hypothesize a substantial chemo-resistant cell population to be present in PDXOs from the heavily pretreated PC2416-DEC tumor tissue, explaining a residual 40% organoid viability (Figure 5B, lower panel). Taken together, our findings indicate that both PDXs and PDXOs are able to reflect patient taxane responses, providing perspectives for individualized drug response profiling and patient stratification.

## 4. Discussion

CRPC continues to be lethal, despite available treatment sequences and treatment combinations. Overall, the AR signaling pathway remains a major oncogenic driver in castration-resistant disease and therapeutic targeting of AR signaling thus remains crucial [1]. Identification of additional clinically actionable targets in CRPC [3,4] opens opportunities for combining AR-targeted with non-AR-targeted therapies. This calls for appropriate AR+ preclinical models, both to test new therapeutic strategies and to study mechanisms of treatment-induced resistance. PCa cells are, however, notoriously difficult to cultivate in vivo and in vitro [7,10,18,53], resulting in a continued underrepresentation of CRPC heterogeneity, specifically regarding heavily pretreated AR+ patients.

While modest in terms of absolute numbers, our newly developed PDXs and PDXOs constitute a highly valuable contemporary addition to former collections. Not only do they faithfully preserve the histomorphological and transcriptomic features of the matching donor tumors, but they also represent actual patient tumors currently seen in the clinic, as confirmed by comparing their genomic and transcriptomic profiles to independent cohorts of CRPC patients. Our PDXs were found to harbor recurrent CNAs in established PCa driver genes and in members of clinically actionable pathways previously identified in CRPC patients [3,4]. These distinct CNA profiles provide a diverse molecular background for future preclinical testing. Of note, a number of CNA differences, such as additional amplifications in driver genes, were identified when comparing the two PDXs originating from the same patient at different stages of disease (PC2416 and PC2146-DEC), making this pair of models uniquely suitable for studies of disease progression. 

At the transcriptomic level, both PDXs and PDXOs resembled CRPC patient tumors with an AR+ adenocarcinoma phenotype and an absence of transcriptional features typical of neuroendocrine or mixed morphologies [30]. This makes our models particularly appealing for in-depth studies of new AR-targeted therapies and AR-driven resistance mechanisms. 

In correspondence with the clinical CRPC setting, we confirmed our CRPC PDXs to thrive in a low androgen environment [52] and observed distinct responses to enzalutamide in PDXOs from enzalutamide-naïve and enzalutamide-pretreated patients. In accordance with previous observations [11,48,49], the reported *AR* overexpression and *AR* variant expression of PC2416-DEC PDX could well be contributing to this enzalutamide resistance, but future studies to explore this hypothesis are required. Additionally, four out of five CRPC PDXs were shown to carry an *AR* amplification, with the *AR* enhancer being included within the amplified region in two of these PDXs. To our knowledge, *AR* enhancer amplification has only been reported in one PCa PDX so far [49].

Continuous updating of existing PCa PDX cohorts has been a long-standing ambition of several international PCa research groups [8,9,10,11], including ours [6,7,42]. Despite this collective expertise, success rates for generating PCa PDXs linger around 10–40% [7,9,10], as illustrated again by the current study. Maximizing exchange, and thus optimal use, of available models is of general importance, but often entails logistic difficulties. PDXOs, which we were able to generate at high success rates, might enhance model transferability within the PCa scientific community. Moreover, PDXOs constitute versatile in vitro models that can be implemented as complementary or substitute models for large-scale preclinical testing. We demonstrated that PDXOs exhibit prominent growth specifically in early generations, allowing for immediate and extensive (targeted) drug testing in highly proliferative organoid cultures. This approach might drastically reduce future animal use, as in vivo testing could well be downsized to validation experiments of promising in vitro hits. Comprehensive verification of morphologic, genetic, transcriptomic and molecular fidelity of PDXOs, next to the exclusion of murine contamination, continues to be paramount. 

To implement the aforementioned approach in future research and eventually maximize translation into clinical trials, the current study first aimed to affirm the potential of PCa PDXs and PDXOs to recapitulate patient treatment responses to standard-of-care therapies. For various cancer types, organoid and PDX drug sensitivities have been heralded as potential predictive biomarkers for treatment responses in matching patients [54,55]. For PCa, however, evidence remains scarce and is limited to single patient case reports [12,49,56,57]. The current study shows that both PCa PDXs and PDXOs can reliably parallel the clinical responses of matching patients. However, we could not investigate the likely intrapatient tumor heterogeneity [58,59], as we only generated PDXs from one sample site per patient. 

This study was also limited to bulk RNA sequencing data and missed genomic data of matching patient tumors. To elucidate potential tumor heterogeneity in our models, further research at the single cell level would be of value [60]. A certain degree of clonal selection is regardless to be expected, as these PDXs were generated from single TURP chips. Patient-derived organoids from metastatic lesions or circulating tumor cells might provide the solution for adequately capturing intrapatient heterogeneity [18,61]. However, in contrast to the majority of solid cancers [62], successes in generating organoids directly from PCa patient tissue have been infrequent [18,53,63,64]. Organoid culture overgrowth by environmental cells, such as fibroblasts or benign epithelial cells, constitutes one of the major obstacles to success [53], which is less frequently reported for other cancer types [62]. We and others [13,53] have not been able to repeat the ground-breaking work from Gao et al. [63] using PCOM [17]. Removing four medium components [18], of which two were also reported by Beshiri et al. [13] to negatively impact organoid growth, has so far led to higher success rates. There is, however, abundant room for further progress in improving current culture efficiency, considering that success rates and optimal growth conditions still vary among different PDX and patient tumors, as also observed by others [13]. 

Using the CRPC PDXs and matching PDXOs from the current study provides an alternative, reliable and unlimited resource for clinically relevant drug targeting in late-stage disease and allows for in-depth investigations on mechanisms of AR-driven resistance and treatment-induced cross-resistance that might guide future patient stratification.

## Data Availability

Data from RNA sequencing and SNP arrays will be made available in a public repository. Additional data generated in this study are available from the corresponding author on reasonable request. PDXs and PDXOs are available from W.M.v.W. under a material transfer agreement with the Erasmus MC. The RNA sequencing data of the Labrecque metastatic CRPC cohort (*n* = 98) were accessed through GEO (accession number GSE126078).

## Supplementary Materials

The following supporting information can be downloaded at: https://www.mdpi.com/article/10.3390/cells11223632/s1, Figure S1: Schematic workflow for culturing PDXOs; Figure S2: Transcriptomic profiles of patients, PDXs and PDXOs; Figure S3: Characteristics of PCa xenografts, related to Figure 2; Figure S4: Establishment of PCa PDXOs, related to Figure 3; Figure S5: AR signaling status of PDXs and PDXOs, related to Figure 4; Figure S6: AR activity in CRPC patients, and matching PDXs and PDXOs. Purity of long-term PDXOs; Figure S7: Taxane responses of CRPC PDXs presented as individual tumor volumes over time; Table S1: Origin of PCa samples for developing subcutaneous PDX models; Table S2: Composition of Prostate Cancer Organoid Medium (PCOM), Adjusted Prostate Cancer Organoid Medium (APCOM) and Prostate Growth Medium (PGM); Table S3: Antibodies used for immunohistochemistry; Table S4: Summary of RNA sequencing and SNP genotyping array datasets; Table S5: Quantitative RT-PCR primers and probes for AR full length and AR variants; Table S6: Matching of short tandem repeat (STR) profiles of CRPC PDXs to the original patient sample and early PDX generations. References [65,66,67,68,69,70,71] are cited in the Supplementary Materials.
