# Supplementary material for: Patient-Derived Xenografts and Organoids Recapitulate Castration-Resistant Prostate Cancer with Sustained Androgen Receptor Signaling
Source: Cells. 2022 Nov 16;11(22):3632. doi: 10.3390/cells11223632 (PMC9688335; doi:10.3390/cells11223632)
Supplement: Supplementary file 1 [file cells-11-03632-s001.zip › cells-1988817-supplementary.pdf]

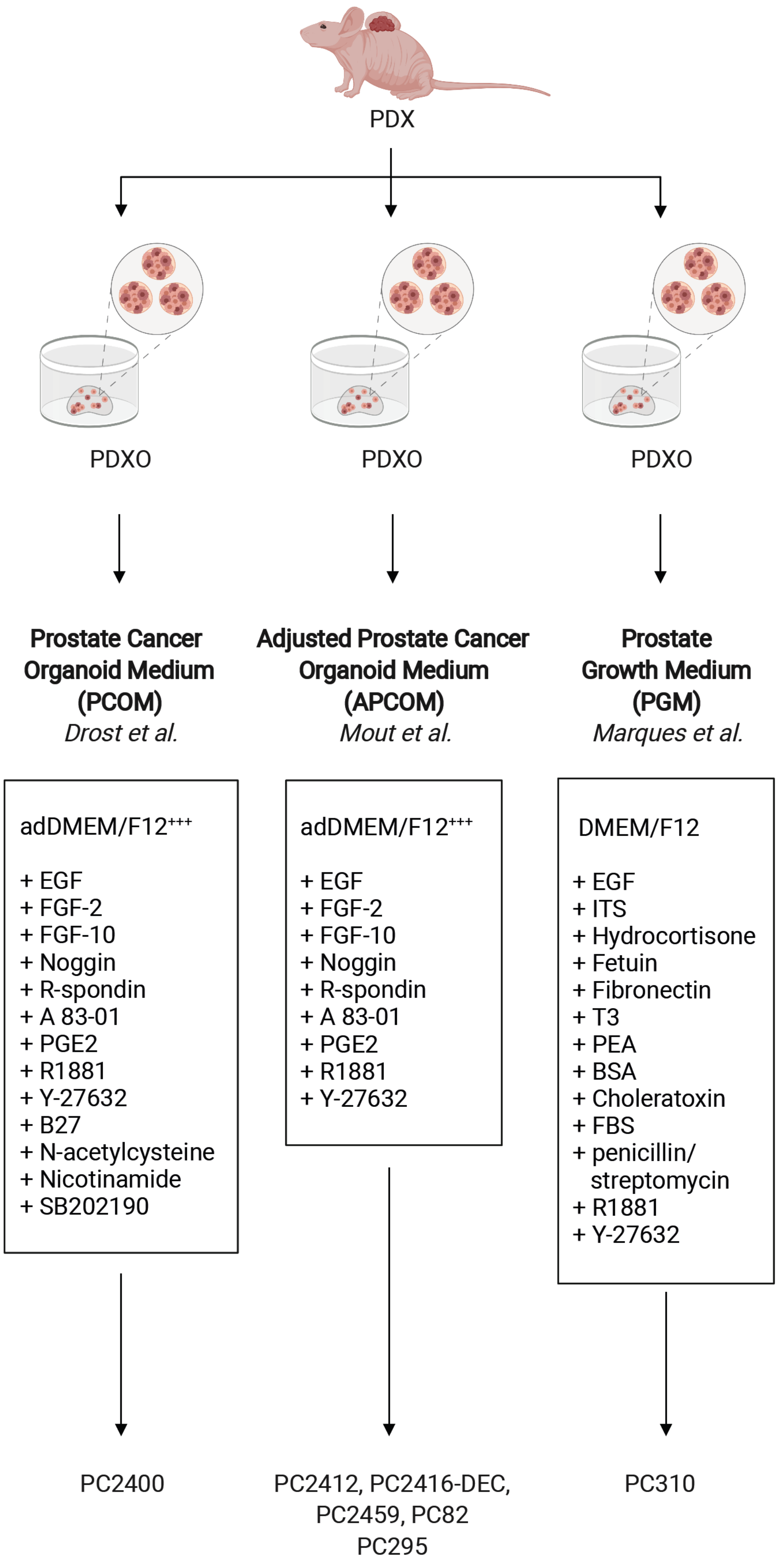

Figure S1. Schematic workflow for culturing PDXOs.
For each PDX tumor, we systematically tested three organoid culture conditions. Different medium compositions are listed in the boxes, and details are provided in Table S2. Most favorable organoid culture conditions were determined for each PDX, based on the combination of successes in organoid initiation, exponential expansion and, in case of CRPC PDXs long-term perpetuation.
Bottom panel summarizes PDXOs that optimally grow in each medium type.
AdDMEM/F12^{+++} = Advanced DMEM/F12 containing 10 mM Hepes, 2 mM L-Glutamine and penicillin/streptomycin.
Drost et al. [17], Mout et al. [18], Marques et al. [19].

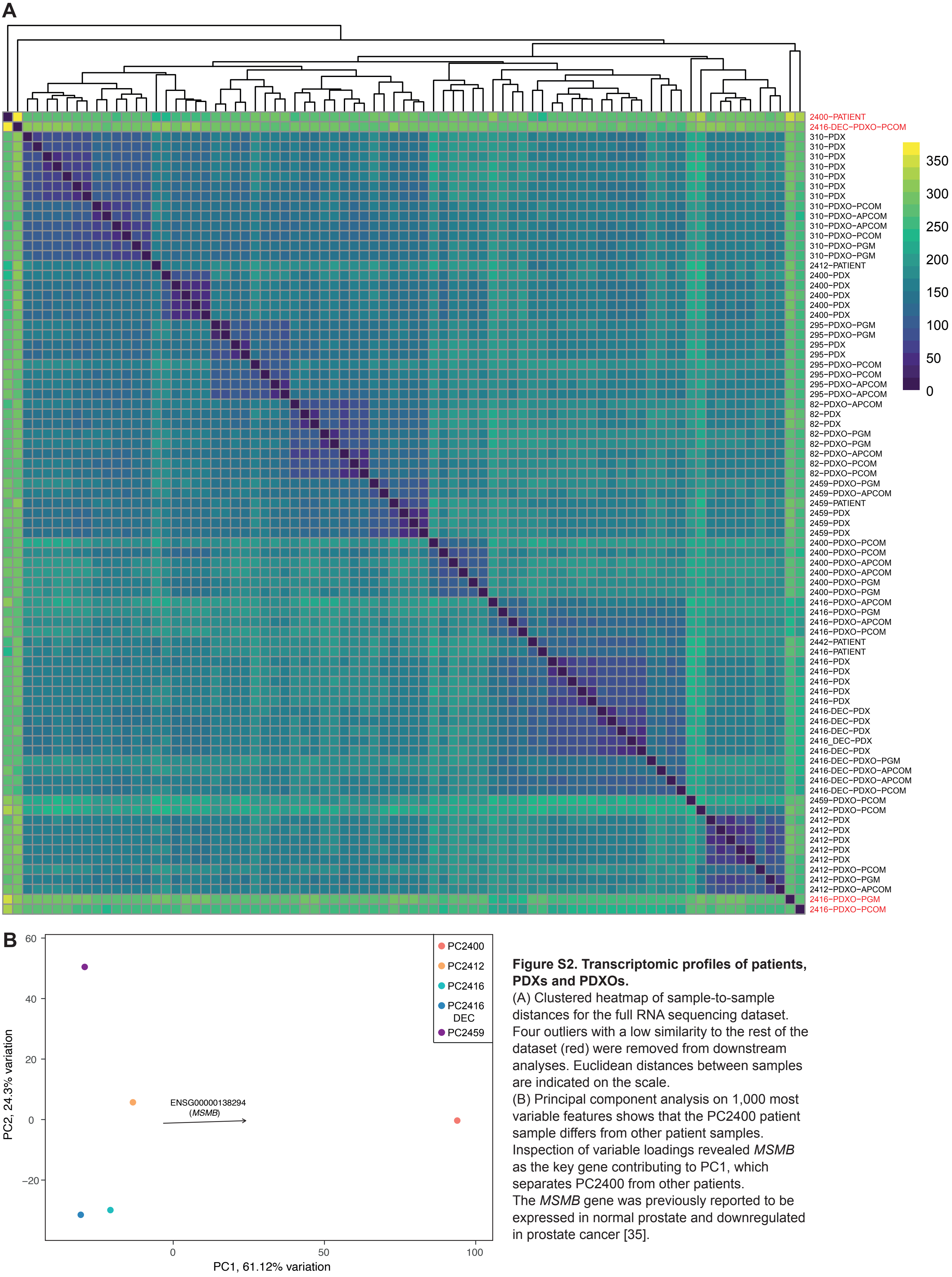

Figure S2. Transcriptomic profiles of patients, PDXs and PDXOs.
(A) Clustered heatmap of sample-to-sample distances for the full RNA sequencing dataset. Four outliers with a low similarity to the rest of the dataset (red) were removed from downstream analyses. Euclidean distances between samples are indicated on the scale.
(B) Principal component analysis on 1,000 most variable features shows that the PC2400 patient sample differs from other patient samples. Inspection of variable loadings revealed *MSMB* as the key gene contributing to PC1, which separates PC2400 from other patients.
The *MSMB* gene was previously reported to be expressed in normal prostate and downregulated in prostate cancer [35].

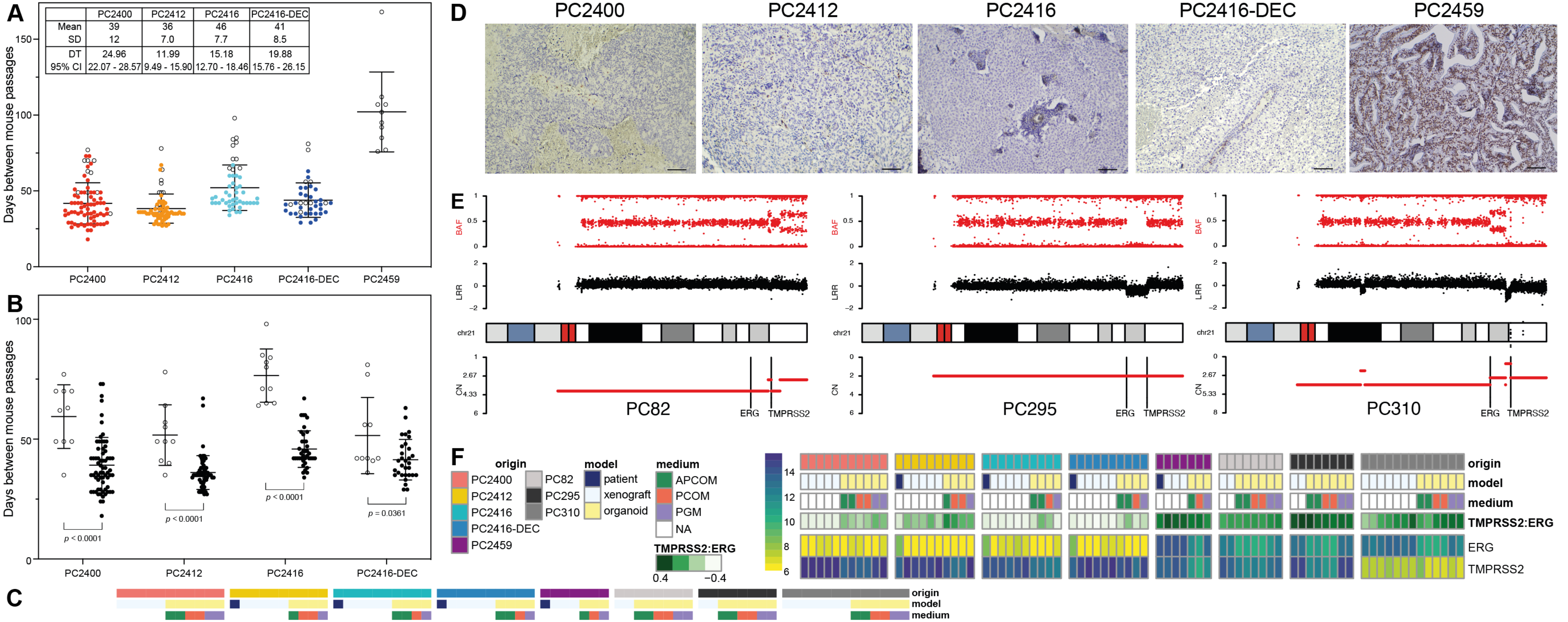

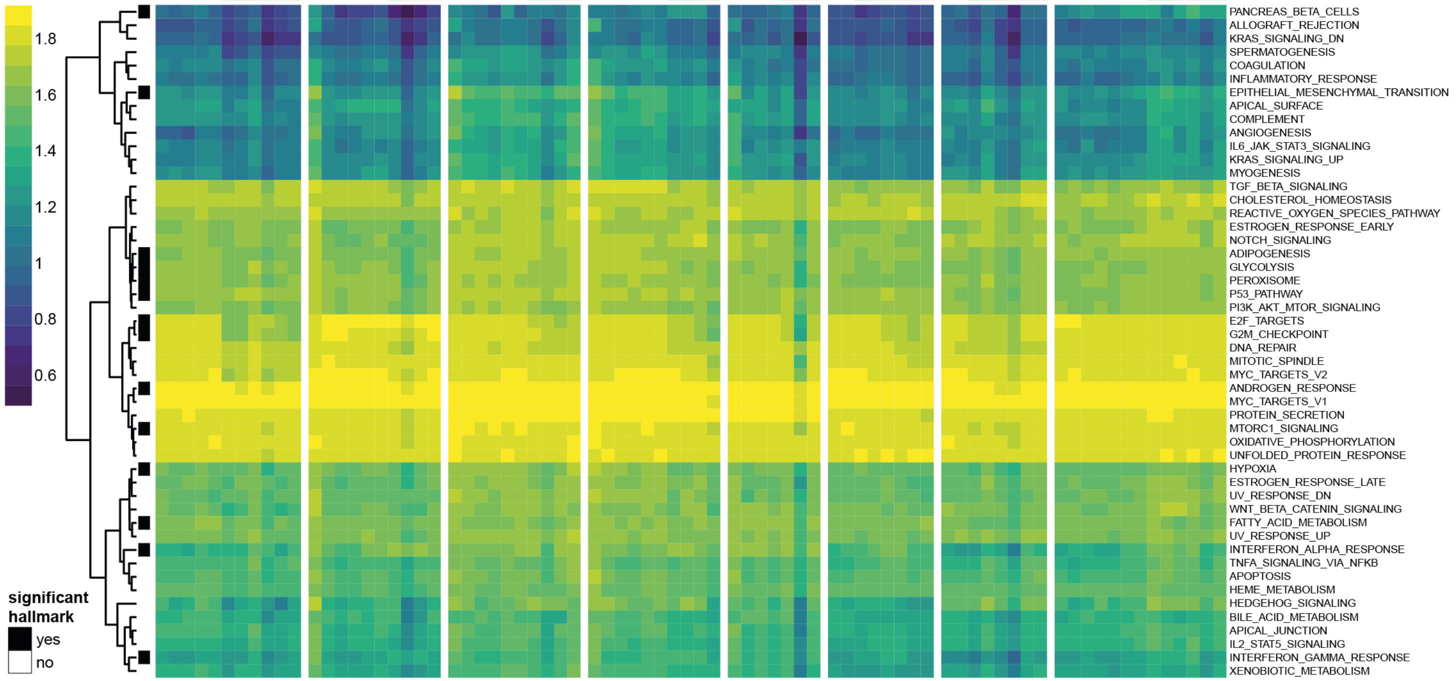

Figure S3. Characteristics of PCa xenografts, related to Figure 2. (A-B) Details on serial transplantations of CRPC PDXs. Each dot represents one mouse passage (mp): open dots indicate mp 1 to 10, solid dots mp >10. Horizontal line depicts mean with error bars of standard deviation (SD), Mann–Whitney U test. PC2459 PDX had undergone 10 mps at time of reporting. The box lists the mean number of days between passages (from mp 10 on), followed by current doubling times (DT).
(C) Pathway activity in original patient samples (n=4), PDXs (n=34) and PDXOs (n=39). The heatmap depicts *singscore* single-sample pathway enrichment for the 50 MSigDB cancer hallmark gene sets. Hallmarks differing significantly between HSPC and CRPC PDXs, based on GSEA, are marked in black.
(D-F) *TMPRSS2-ERG* gene fusion status of PCa PDXs. (D) ERG staining in CRPC PDXs. Scale bars indicate 100 µm. (E) Chromosome 21 copy number profile of HSPC PDXs known to harbor a *TMPRSS2-ERG* fusion [46]. B-allele frequency (BAF, red) and log R ratio (LRR, black) are displayed (top panel), along with the total copy number (CN) inferred by ASCAT (bottom panel). (F) Heatmap of *TMPRSS2* and *ERG* gene expression across the entire dataset. Expression levels are shown in normalized vst counts as determined by RNA sequencing. GSVA single-sample enrichment scores for a set of genes upregulated in tumors harboring the *TMPRSS2-ERG* fusion (TMPRSS2:ERG_signature_UP [25,65]) are depicted in ranges of green.

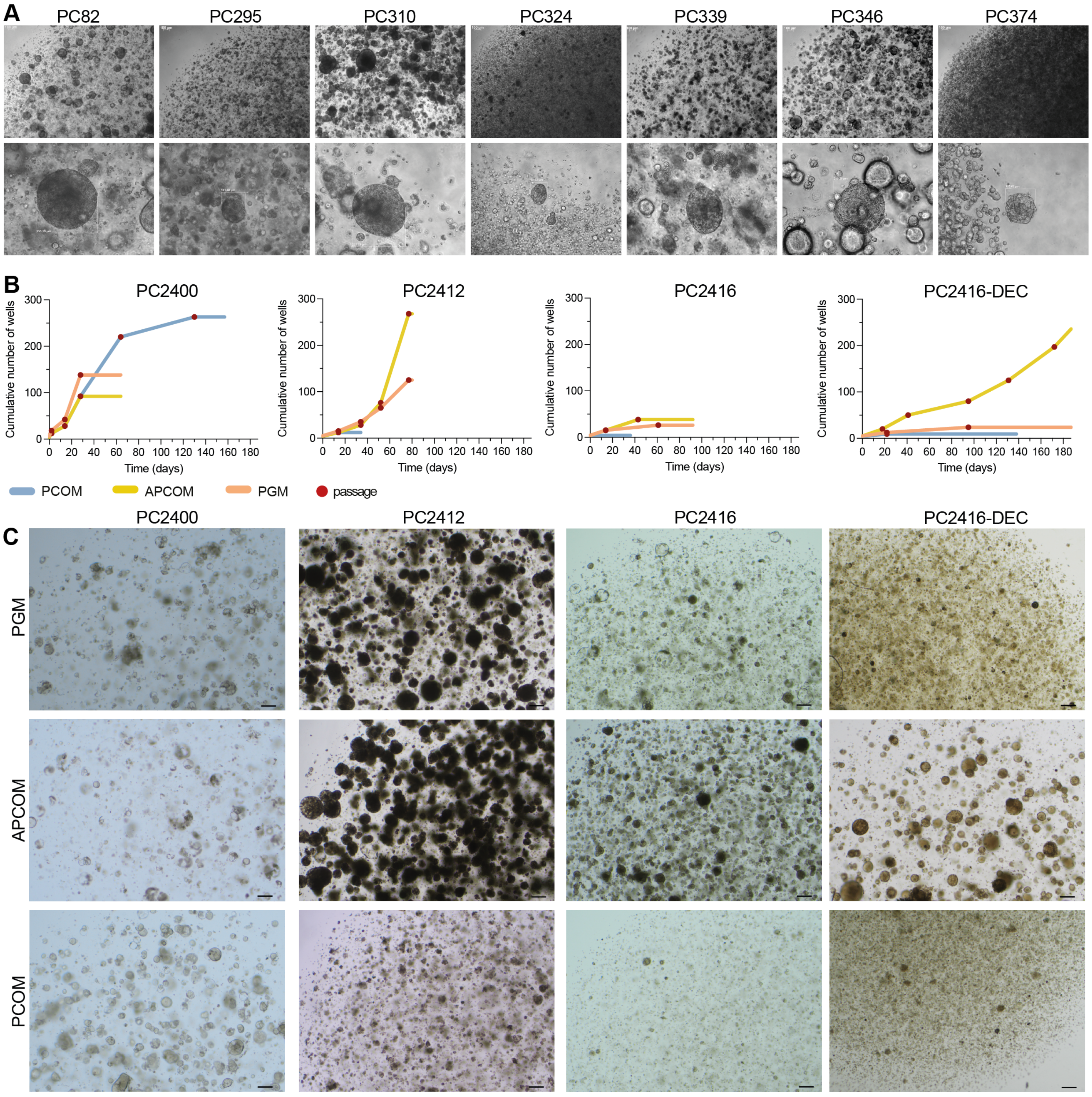

Figure S4. Establishment of PCa PDXOs, related to Figure 3.
(A) Representative images of short-term PDXOs from previously published PDXs [6,42]. Top row 50x magnification, bottom row 200x magnification.
(B-C) Validation of most favorable organoid culture conditions for long-term culturing of CRPC PDXOs as determined in Figure 3C-D. APCOM was confirmed to be the preferred medium for PC2412 and PC2416-DEC, PCOM for PC2400. PC2459 PDX, for which tumor material was still scarce during early PDX development, is not included.
(B) The cumulative number of wells with PDXOs for each PDX during long-term *in vitro* perpetuation. Each passage is marked with a red dot. Different culture media are indicated by color: blue represents PCOM, yellow APCOM and orange PGM. PC2416 PDXO cultures were discontinued early due to murine contamination, PC2412 due to fungal infection.
(C) Representative images of PDXOs cultured in the three distinct media. Images were acquired at day 38, day 34, day 21, and day 78 for PC2400, PC2412, PC2416 and PC2416-DEC, respectively. Scale bars equal 100 µm.

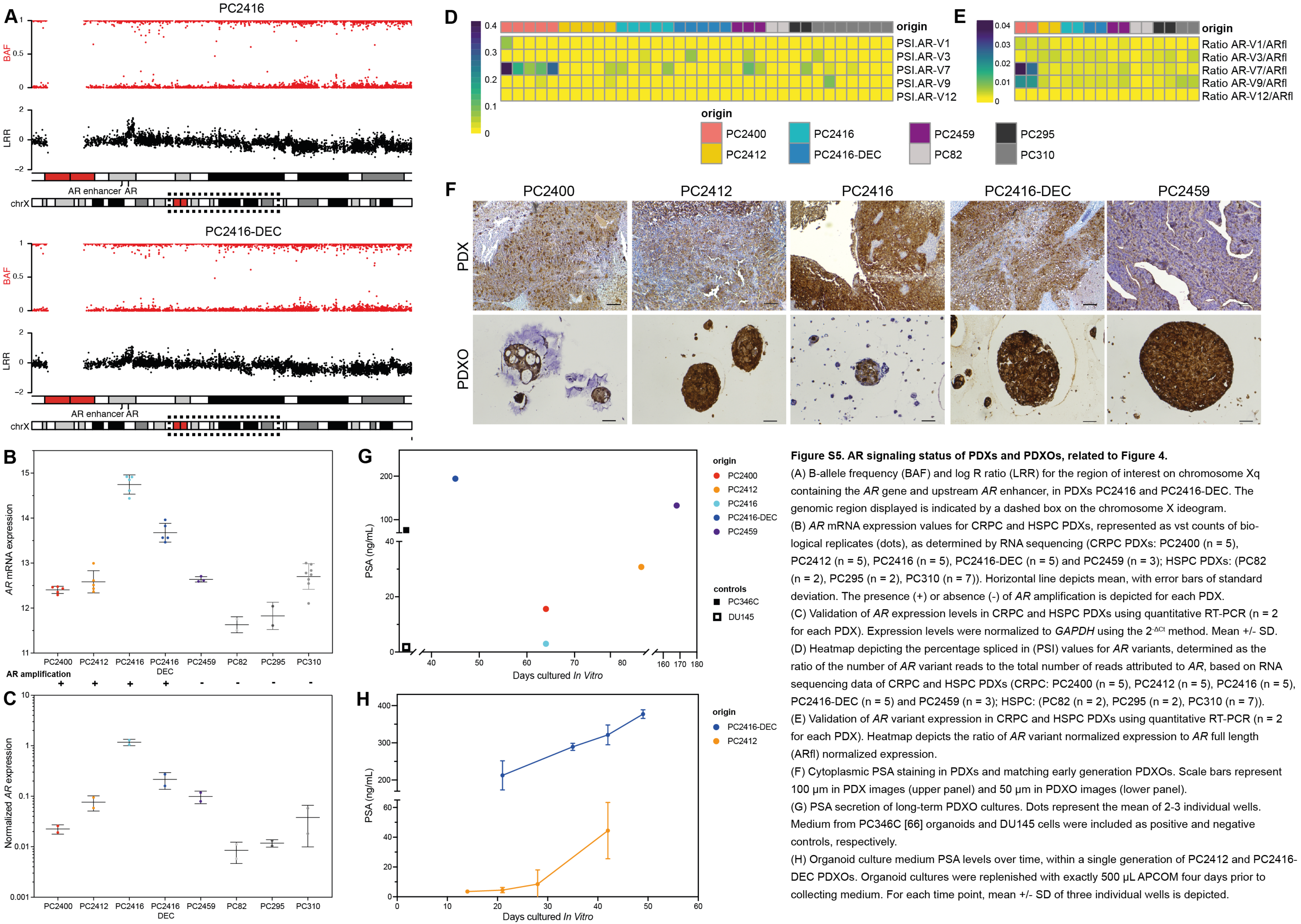

Figure S5. AR signaling status of PDXs and PDXOs, related to Figure 4.

(A) B-allele frequency (BAF) and log R ratio (LRR) for the region of interest on chromosome Xq containing the *AR* gene and upstream *AR* enhancer, in PDXs PC2416 and PC2416-DEC. The genomic region displayed is indicated by a dashed box on the chromosome X ideogram.

(B) *AR* mRNA expression values for CRPC and HSPC PDXs, represented as vst counts of biological replicates (dots), as determined by RNA sequencing (CRPC PDXs: PC2400 (n = 5), PC2412 (n = 5), PC2416 (n = 5), PC2416-DEC (n = 5) and PC2459 (n = 3); HSPC PDXs: (PC82 (n = 2), PC295 (n = 2), PC310 (n = 7)). Horizontal line depicts mean, with error bars of standard deviation. The presence (+) or absence (-) of *AR* amplification is depicted for each PDX.

(C) Validation of *AR* expression levels in CRPC and HSPC PDXs using quantitative RT-PCR (n = 2 for each PDX). Expression levels were normalized to *GAPDH* using the $2^{-\Delta Ct}$ method. Mean +/- SD.

(D) Heatmap depicting the percentage spliced in (PSI) values for *AR* variants, determined as the ratio of the number of *AR* variant reads to the total number of reads attributed to *AR*, based on RNA sequencing data of CRPC and HSPC PDXs (CRPC: PC2400 (n = 5), PC2412 (n = 5), PC2416 (n = 5), PC2416-DEC (n = 5) and PC2459 (n = 3); HSPC: (PC82 (n = 2), PC295 (n = 2), PC310 (n = 7)).

(E) Validation of *AR* variant expression in CRPC and HSPC PDXs using quantitative RT-PCR (n = 2 for each PDX). Heatmap depicts the ratio of *AR* variant normalized expression to *AR* full length (ARfl) normalized expression.

(F) Cytoplasmic PSA staining in PDXs and matching early generation PDXOs. Scale bars represent 100 µm in PDX images (upper panel) and 50 µm in PDXO images (lower panel).

(G) PSA secretion of long-term PDXO cultures. Dots represent the mean of 2-3 individual wells. Medium from PC346C [66] organoids and DU145 cells were included as positive and negative controls, respectively.

(H) Organoid culture medium PSA levels over time, within a single generation of PC2412 and PC2416-DEC PDXOs. Organoid cultures were replenished with exactly 500 µL APCOM four days prior to collecting medium. For each time point, mean +/- SD of three individual wells is depicted.

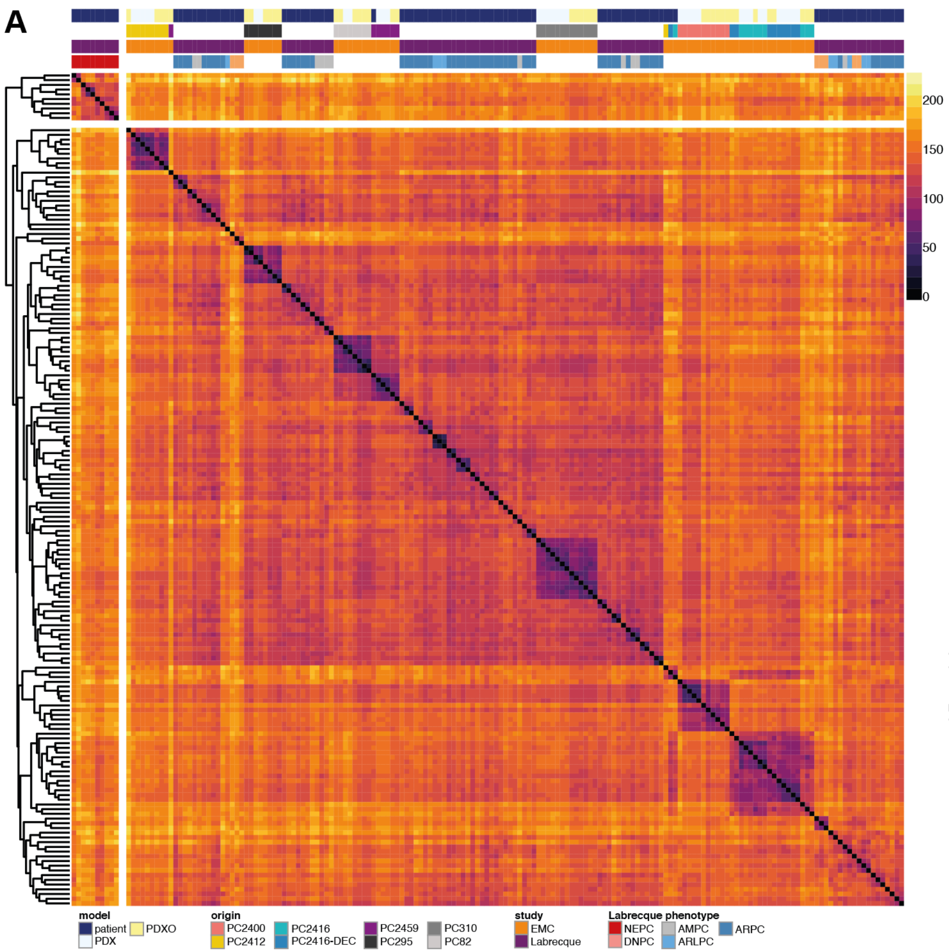

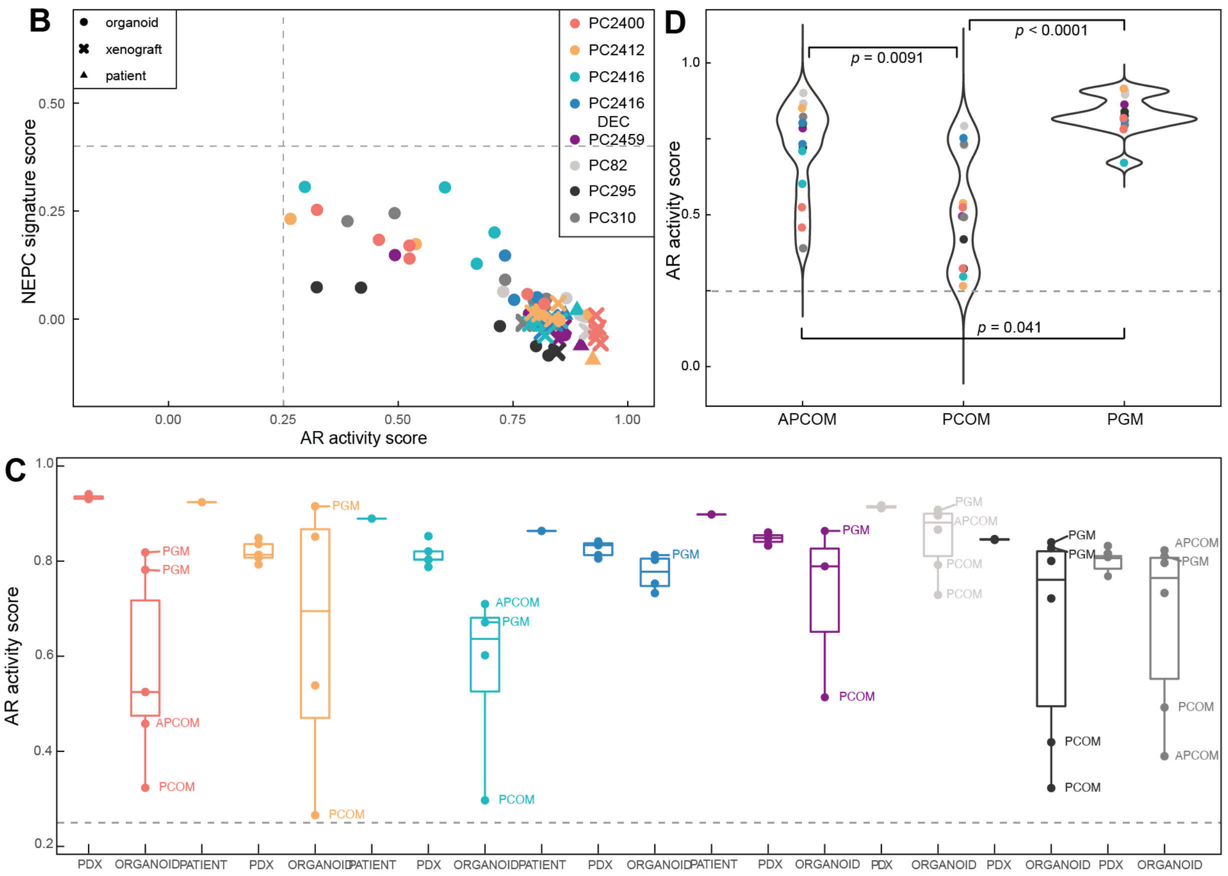

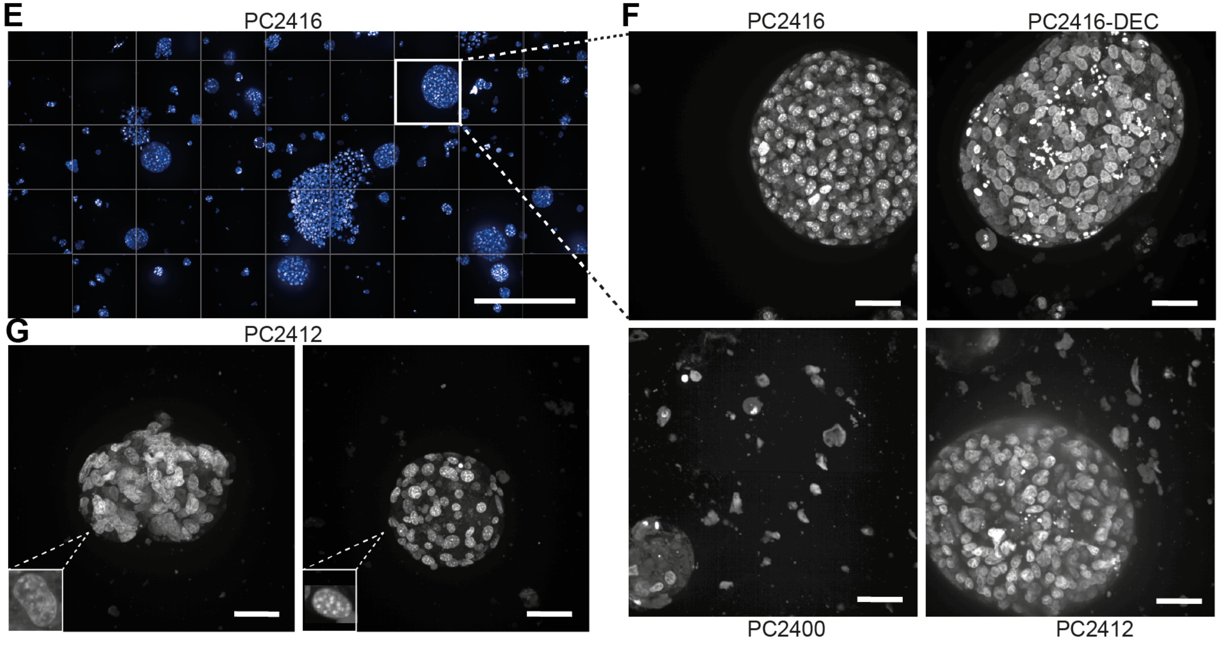

Figure S6. AR activity in CRPC patients, and matching PDXs and PDXOs. Purity of long-term PDXOs.
(A) Clustered heatmap of sample-to-sample distances for the full RNA sequencing dataset pooled with the patient cohort from Labrecque et al. [30]. Euclidean distances between samples are indicated on the scale as a measure of (dis)similarity. The pooled data comprises patients (n=4;blue), PDXs (n=34;white) and PDXOs (n=39;yellow) from this study (EMC, orange) and patients (n=98) from the Labrecque cohort (purple). EMC samples are labelled according to PDX origin. Patient samples from Labrecque et al. are labelled according to CRPC phenotype as reported by the authors: tumors with small cell or neuroendocrine gene expression without AR activity (NEPC, red), double-negative (AR-/NE-) tumors (DNPC, pink), amphicrine tumors co-expressing AR and NE genes (AMPC, grey), AR-low tumors (ARLPC, light blue) and AR-high tumors (ARPC, dark blue). The patient samples most divergent from our preclinical models belong to the NEPC phenotype and form a separate cluster from the remainder of the dataset.
(B) Landscape plot of AR activity and NE signature scores in patients (n=4), PDXs (n=34) and PDXOs (n=39). Thresholds separating samples with low versus high scores are marked with dotted lines.
(C) Boxplots summarizing AR activity scores of (B) per patient-of-origin. Boxes depict first and third quartiles, with median represented as a solid line. Outliers among PDXOs are denoted by the medium used for culturing. Sample color coding is identical to (B) and (D).
(D) AR activity scores of PDXOs according to medium type. PCOM was associated with significantly lower AR scores compared to APCOM and PGM; no significant difference in AR scores was observed between APCOM and PGM after correcting for multiple testing (Wilcoxon signed rank test).
(E-G) Live-cell imaging of Hoechst stained long-term PDXO cultures to identify murine contamination. (E) Overview of a single well of PC2416-derived organoids after six months of *in vitro* propagation. The culture consists entirely of murine organoids as illustrated by the presence of pericentromeric heterochromatin clusters, or chromocenters, a distinctive feature of murine cells [67,68]. Scale bar indicates 500 µm. (F) Detail of a single organoid from each PDX, propagated for at least two months in optimal medium. Identification of murine organoids only in PC2416 PDXO cultures; organoids from other PDXs demonstrate human origin. Scale bars equal 50 µm. (G) Detail of long-term PC2412 organoids, propagated for 65 days in PGM. PDXO of human origin on the left and murine origin on the right side. Scale bars 50 µm.

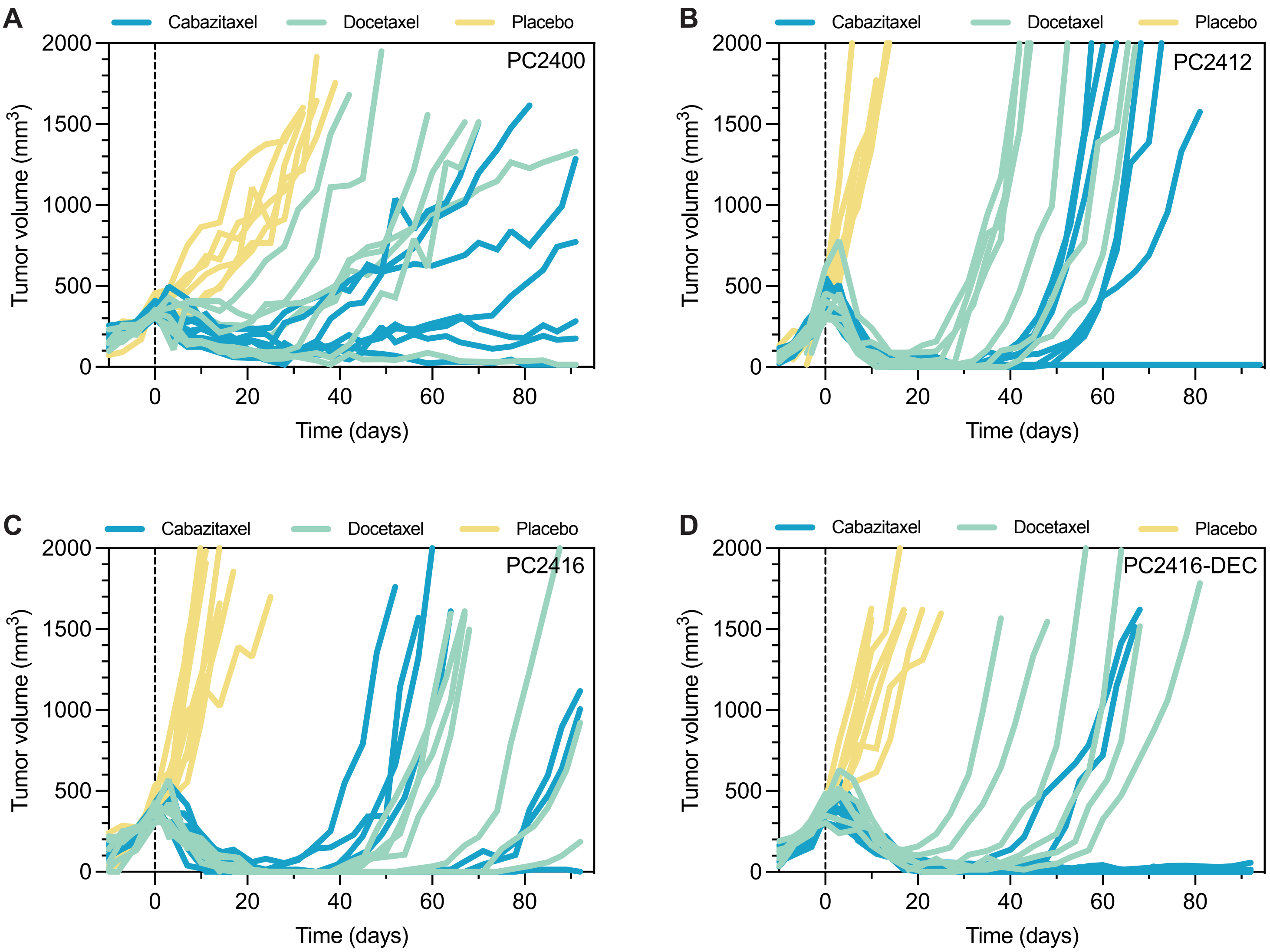

Figure S7. Taxane responses of CRPC PDXs presented as individual tumor volumes over time.
A single dose of 33 mg/kg cabazitaxel (blue), 33 mg/kg docetaxel (green), or placebo (yellow) was administered intravenously at day 0, when tumors passed a volume of 300 mm^3. Tumor volumes were measured twice a week. Follow-up was set to 90 days after treatment or tumor volumes exceeding 1500 mm^3.
(A) Individual tumor volumes of PC2400 bearing mice treated with cabazitaxel (n=8), docetaxel (n=7), or placebo (n=6).
(B) Individual tumor volumes of PC2412 bearing mice treated with cabazitaxel (n=7), docetaxel (n=8), or placebo (n=7).
(C) Individual tumor volumes of PC2416 bearing mice treated with cabazitaxel (n=7), docetaxel (n=7), or placebo (n=8).
(D) Individual tumor volumes of PC2416-DEC bearing mice treated with cabazitaxel (n=8), docetaxel (n=8), or placebo (n=7).

SUPPLEMENTARY TABLES

SAMPLE ORIGIN	SUBCUTANEOUS IMPLANTATIONS n (%)	TUMOR TAKE n (%)	ESTABLISHED PDXS n (%)
**TURP**	31 (81.58)	8 (88.89)	5 (100)
**RP**	1 (2.63)	0 (0)	0 (0)
**Prostate biopsy**	1 (2.63)	0 (0)	0 (0)
**Lymph node metastasis**	4 (10.53)	1 (11.11)	0 (0)
**Bone metastasis**	1 (2.63)	0 (0)	0 (0)
	**38 (100)**	**9 (100)**	**5 (100)**

Table S1. Origin of PCa samples for developing subcutaneous PDX models. Tumor take was defined as successful engraftment followed by one to two mouse transplantations; established PDXs were defined by >5 mouse passages. TURP: transurethral resection of the prostate; RP: radical prostatectomy.

CELL CULTURE REAGENT	SUPPLIER (catalogue number)	CONCENTRATION	MEDIUM
Advanced DMEM/F12 (AdDMEM/F12)[a]	Thermo Fisher Scientific, Waltham, Massachusetts, USA (cat. no. 12634010)	-	PCOM, APCOM
DMEM/F12	Lonza, Basel, Switzerland (cat. no. BE12-719F)	-	PGM
Hepes[a]	ThermoFisher Scientific (cat. no. 15630056)	10 mM	PCOM, APCOM
L-Glutamine[a]	Lonza (cat. no. BE17-605E)	2 mM	PCOM, APCOM
Penicillin/Streptomycin[a]	Lonza (cat. no. DE17-602E)	100 U/mL	PCOM, APCOM, PGM
Epidermal Growth Factor (EGF)	Sigma-Aldrich, Saint Louis, Missouri, USA (cat. no. E9644)	20 ng/mL<br>10 ng/mL	PCOM, APCOM,<br>PGM
Fibroblast Growth Factor 2 (FGF-2)	R&D Systems, Minneapolis, Minnesota, USA (cat. no. 233-FB-025)	5 ng/mL	PCOM, APCOM
Fibroblast Growth Factor 10 (FGF-10)	PeproTech, Cranbury, New Jersey, USA (cat. no. 100-26)	10 ng/mL	PCOM, APCOM
Noggin	Conditioned medium from Hek293T-Noggin-Fc[1]	-	PCOM, APCOM
R-Spondin	Conditioned media from Hek293T-hRSpo1[2]	-	PCOM, APCOM
A 83-01	Tocris Bioscience, Abingdon, UK (cat. no. 2939)	500 nM	PCOM, APCOM
Prostaglandin E2 (PGE2)	Tocris Bioscience (cat. no. 2296)	1 µM	PCOM, APCOM
R1881	Sigma-Aldrich (cat. no. R0908)	0.1 nM	PCOM, APCOM, PGM
Y-27632 dihydrochloride	Adipogen, San Diego, California, USA (cat. no. AG-CR1-3564-M025)	10 µM	PCOM, APCOM, PGM
B27 Supplement (50 X)	ThermoFisher Scientific (cat. no. 17504-044)	1 X	PCOM
N-Acetyl-L-cysteine	Sigma-Aldrich (cat. no. A9165)	1.25 mM	PCOM
Nicotinamide	Sigma-Aldrich (cat. no. N0636)	10 mM	PCOM
SB 202190	Sigma-Aldrich (cat. no. S7067)	10 µM	PCOM
Insulin-Transferrin-Selenium (ITS) (100 X)	ThermoFisher Scientific (cat. no. 41400045)	1 X	PGM
Hydrocortisone	Sigma-Aldrich (cat. no. H0888)	0.5 µg/mL	PGM
Fetuin	Sigma-Aldrich (cat. no. F3385)	20 µg/mL	PGM
Fibronectin	ThermoFisher Scientific (cat. no. 33016015)	100 ng/mL	PGM
Triiodo-L-thyronine (T3)	Sigma-Aldrich (cat. no. T6397)	0.6 ng/mL	PGM
Phosphorylethanolamine (PEA)	Sigma-Aldrich (cat. no. P0503)	100µM	PGM
Bovine Serum Albumin Fraction V (BSA)	Roche Diagnostics, Mannheim, Germany (cat. no. 10735094001)	0.01% (w/v)	PGM
Cholera Toxin	Sigma-Aldrich (cat. no. C8052)	50 ng/mL	PGM
FBS	ThermoFisher Scientific (cat. no.10270106)	2% (v/v)	PGM
[a]Components for adDMEM/F12^{+++}, [1]Heijmans et al. [69], [2]Kim et al. [70]			

Table S2. Composition of Prostate Cancer Organoid Medium (PCOM), Adjusted Prostate Cancer Organoid Medium (APCOM) and Prostate Growth Medium (PGM).

PRIMARY ANTIBODY	SUPPLIER (catalogue number)	DILUTION FOR TISSUE	DILUTION FOR ORGANOIDS
Androgen Receptor (SP107) Rabbit Monoclonal Antibody	Cell Marque, Rocklin, California, USA (cat. no. 200R-16)	1:200	1:200
Polyclonal Rabbit Anti-Human Prostate-Specific Antigen	Dako Agilent, Santa Clara, California, USA (cat. no. A0562)	1:500	1:5000
ERG (EPR3864) Rabbit Monoclonal Antibody	Roche Diagnostics, Mannheim, Germany (cat. no. 790-4576)	1	1

SECONDARY ANTIBODY	SUPPLIER (catalogue number)
Goat Anti-Rabbit Immunoglobulins/HRP	Dako Agilent, Santa Clara, California, USA (cat. no.K4003)

Table S3. Antibodies used for immunohistochemistry.

		RNA sequencing			SNP arrays
		PATIENT TUMOR n	PDX n	PDXO n	PDX n
**THIS STUDY**	PC2400	1	5	6	1
	PC2412	1	5	4	1
	PC2416	1	5	6	1
	PC2416-DEC	1	5	5	1
	PC2459	1	3	3	1
	PC82	0	2	6	1
	PC295	0	2	6	1
	PC310	0	7	6	1
	**TOTAL**	**5**	**34**	**42**	**8**
**EXTERNAL DATASET**	Labrecque et al. [30] (GSE126078)	98			

Table S4. Summary of RNA sequencing and SNP genotyping array datasets.

PRIMERS AND PROBES FOR AR FULL LENGTH AND AR VARIANTS		
**AR full length**	FORWARD	5'-CTGCTCAAGACGCTTCTA-3'
	REVERSE	5'-ATCATTTCCGGAAGTCCA-3'
	PROBE	5'-TCCGTGCAGCCTATTGCGAG-3'
**AR-V1**	FORWARD	5'-GTCGTCTTCGGAAATGTTA-3'
	REVERSE	5'-TGAGAGTCTGAAGGTAGTCT-3'
	PROBE	5'-ACTCTGGGAGCAGCTGTTGTT-3'
**AR-V3**	FORWARD	5'-CTTCTGGGTGTCACTATG-3'
	REVERSE	5'-CTTGGGGTTAGTGTCTGA-3'
	PROBE	5'-AGAGCCGCTGAAGGATTTTTCAGA-3'
**AR-V7**	FORWARD	5'-GTCCATCTTGTCGTCTTC-3'
	REVERSE	5'-GCAAGTCAGCCTTTCTTCA-3'
	PROBE	5'-GGGAGAAAAATTCCGGGTTGGC-3'
**AR-V9**	FORWARD	5'-GTCTTCGGAAATGTTATGAA-3'
	REVERSE	5'-CTGCGTGTTTTTCCCTTAG-3'
	PROBE	5'-CTGGGAGACAACTTACCTGAGC-3'
**AR-V12**	FORWARD	5'-CTTTGCAGCCTTGCTCTCTA-3'
	REVERSE	5'-CTTGCCTGATTGCGAGAGAG-3'
	PROBE	5'-ACACGTGGTCAAGTGGGCCA-3'

Table S5. Quantitative RT-PCR primers and probes for AR full length and AR variants.

	% MATCH TO PATIENT	% MATCH TO EARLY PDX	% MATCH TO PC2416 PDX MP3
**PC2400**			
PDX MP5	100	-	-
PDX MP65	100	100	-
**PC2412**			
PDX MP3	92.59	-	-
PDX MP54	92.86	96.15	-
**PC2416***			
PDX MP3	96.67	-	-
PDX MP36	94.92	98.25	98.25
**PC2416-DEC***			
PDX MP4	98.36	-	98.31
PDX MP30	93.33	94.92	96.55
**PC2459**	-	-	-
PDX MP2	100	-	-

Table S6. Matching of short tandem repeat (STR) profiles of CRPC PDXs to original patient sample and early PDX generations [71]. Percent match in STR profiles based on 15 autosomal STR loci plus amelogenin as compared to the original patient tissue, matching early generation PDX (mouse passage 3-5) and early PC2416 PDX. PC2459 PDX was only recently established, hence we exclusively report on the early generation PDX. MP: mouse passage; * common patient origin; - not applicable
